# Supplementary material for: Erythroid-intrinsic activation of TLR8 impairs erythropoiesis in inherited anemia
Source: Nat Commun. 2024 Jul 6;15:5678. doi: 10.1038/s41467-024-50066-w (PMC11227506; doi:10.1038/s41467-024-50066-w)
Supplement: Supplementary file 1 — Supplementary Information [file 41467_2024_50066_MOESM1_ESM.pdf]

Supplementary information

Erythroid-intrinsic activation of TLR8 impairs erythropoiesis in inherited anemia

Jing Liang[1, 2#], Yang Wan[1, 2, 3#], Jie Gao[1, 2#], Lingyue Zheng[1, 2#], Jingwei Wang[1, 2], Peng Wu[1, 2], Yue Li[1, 2], Ding Wang[1, 2], Bingrui Wang[1, 2], Yige Ma[1, 2], Biao Shen[1, 2], Xue Lv[1, 2], Di Wang[1, 2], Na An[4], Xiaoli Ma[5], Guangfeng Geng[4], Jingyuan Tong[1, 2], Jinhua Liu[1, 2], Guo Chen[4], Meng Gao[6], Ryo Kurita[7], Yukio Nakamura[7], Ping Zhu[1,2], Hang Yin[8], Xiaofan Zhu[1, 2, 3*], Lihong Shi[1,2*]

Supplementary figures

Supplementary Figure 1

a

Peripheral blood

Parameters	Normal range	Proband
Neutrophil ($\times 10^9$/L)	2-7	8.96
Monocyte ($\times 10^9$/L)	0.12-1	2.87
Lymphocyte ($\times 10^9$/L)	0.8-4	19.58
MCHC (g/L)	320-360	317
MCH (pg)	27-34	30.1
C-reactive protein (mg/L)	0-10	8.5

b

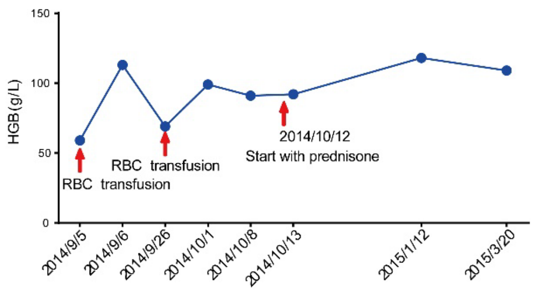

c

d

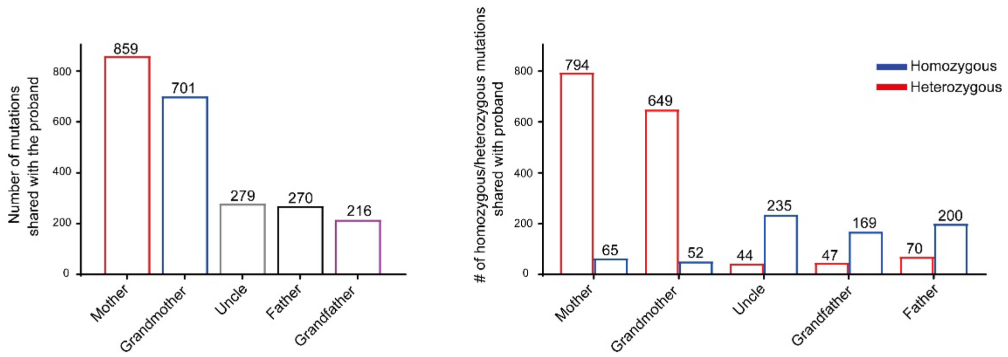

Supplementary Figure 1. The of Hb curve of the proband and the mutations analysis. (a) Other parameters of PB of the proband. (b) The Hb curve over time under steroids treatment and red blood cell transfusion. (c) Numbers of mutations shared by the proband and his mother, grandmother, father, grandfather, and uncle. (d) The numbers of homozygous and heterozygous mutations shared between the proband and his mother, grandmother, father, grandfather, or uncle.

Supplementary Figure 2

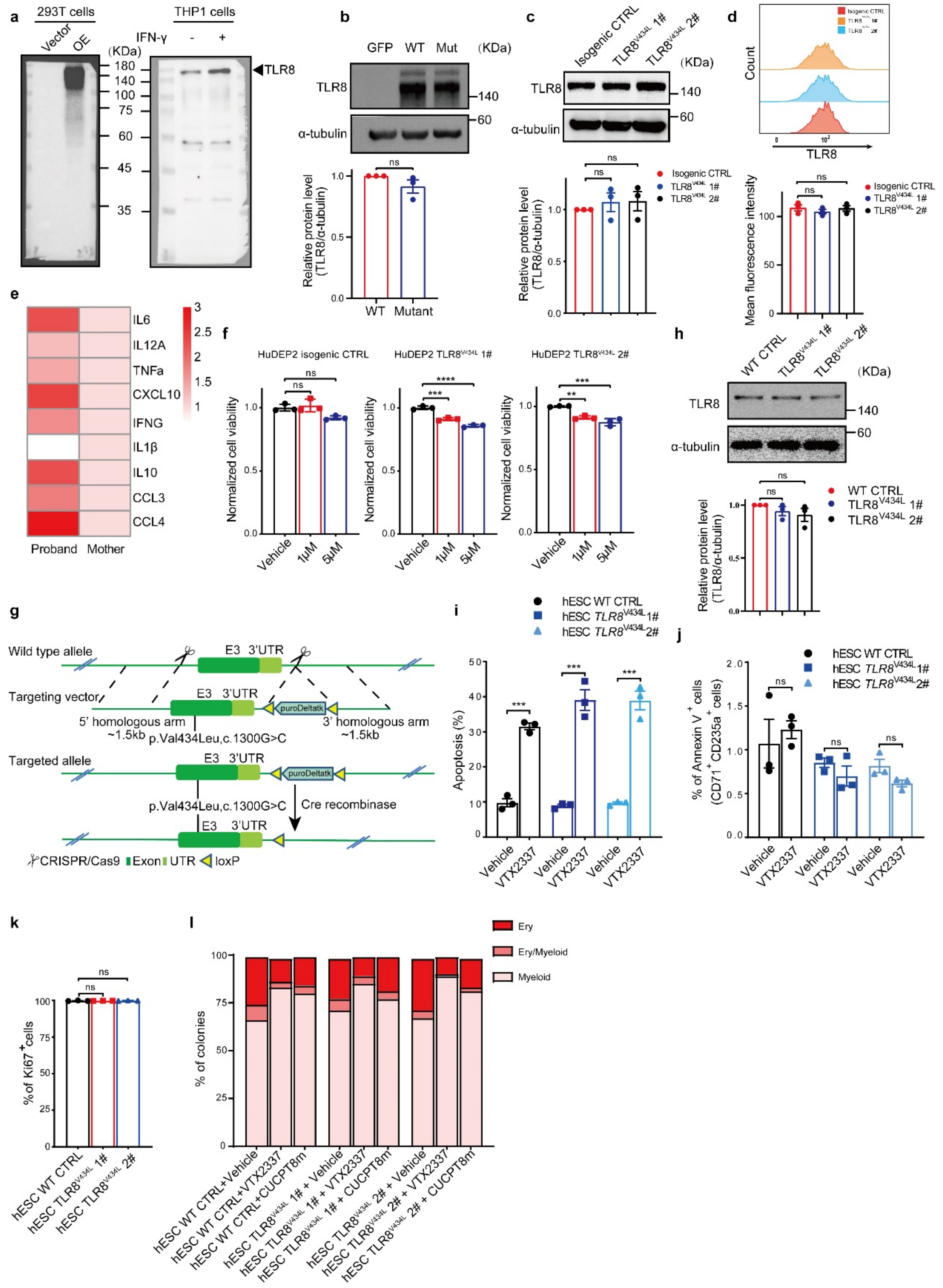

Supplementary Figure 2. The *TLR8^{V434L}* mutation is a GOF mutation and impairs erythropoiesis. (a) Representative immunoblotting of TLR8 in HEK293T cells expressing TLR8 and THP1 cells treated with or without 20 ng/ml IFNγ. (b) Representative

immunoblotting of TLR8 in HEK293T cells transfected with GFP/ *TLR8*^{WT} / *TLR8*^{mutant} plasmids for 24h followed by VTX2337 treatment for 24h and the quantitation (n = 3 biologically independent experiments). (c) Representative immunoblotting of TLR8 in differentiated THP1 cells and the quantitation (n = 3 biologically independent experiments). (d) Representative flow cytometry of TLR8 in differentiated THP1 cells and the quantitation (n = 3 biologically independent experiments). (e) Relative expression of the downstream targets of TLR8 in primary PBMNs from the proband and his mother. (f) Cell viability of isogenic CTRL and *TLR8*^{V434L} mutant HuDEP2 cells treated with various concentrations of VTX2337 for 48h. The viability of cells was normalized to cells without treatment (n = 3 biologically independent experiments). (g) Schematic illustrating CRISPR/Cas9-mediated knock-in strategy of the mutant hESCs. (h) Representative immunoblotting of TLR8 in CD45$^+$ hESCs-derived HSPCs and the quantitation (n = 3 biologically independent experiments). (i) The analysis of total apoptosis in WT and TLR8 mutant cells on day 8 of erythroid differentiation (n = 3 biologically independent experiments). (j) The analysis of Annexin V$^+$ cells in CD71$^+$CD235a$^+$ population with/without VTX2337 treatment on day 8 of erythroid differentiation (n = 3 biologically independent experiments). (k) The percentage of Ki67$^+$ cells in colonies collected on day 14. (l) The percentage of CFUs formed by 12000 CD45$^+$ HSPCs from WT and mutant ESCs treated with vehicle, 10 μM VTX2337 or 10 μM CUCPT8m (n = 3 biologically independent experiments). Data are mean ± SEM; statistical significance was determined using the unpaired two-tailed Student's t-test for two groups comparison or one-way ANOVA for multiple comparisons. ***P* < 0.01, ****P* < 0.001, *****P* < 0.0001, ns = not significant.

Supplementary Figure 3

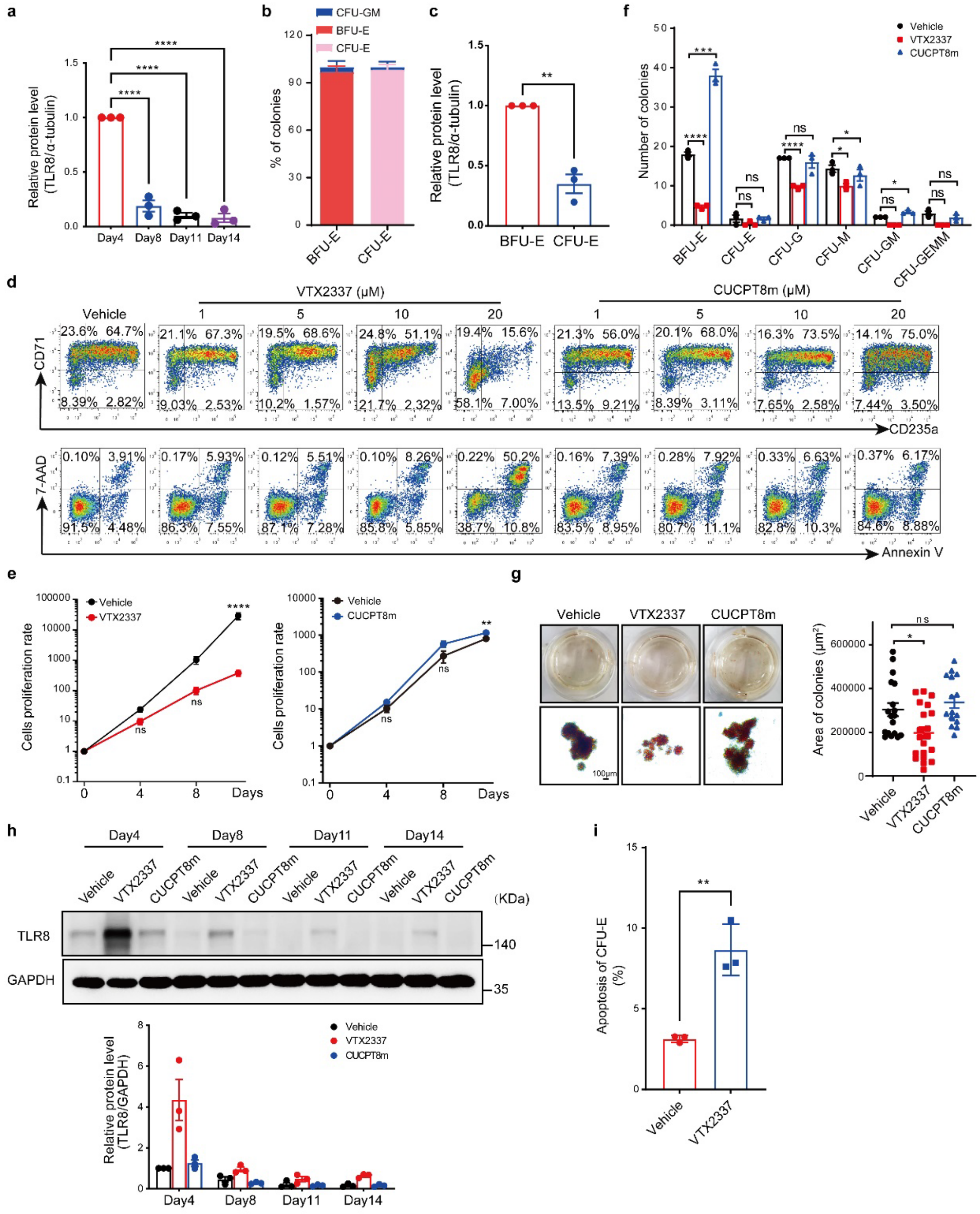

Supplementary Figure 3. TLR8 activation impairs erythropoiesis. (a) The quantitation of TLR8 in CD34$^+$ on days 4, 8, 11, and 14 (n = 3 biologically independent experiments). (b) Frequencies of colonies generated by sorted erythroid progenitors on day 5 of erythroid differentiation (n = 4 biologically independent experiments). (c) The quantitation of TLR8 in BFU-E and CFU-E cells (n = 3 biologically independent experiments). (d) The effects of VTX2337 and CUCPT8m on erythroid differentiation and apoptosis of day 11 differentiated erythroid cells. Cultures were supplemented with VTX2337 and CUCPT8m at various

concentrations from days 8 to 11 of differentiation. (e) Cumulative proliferation curves of $CD34^+$ cells subjected to 10 μM VTX2337 or 10 μM CUCPT8m treatment from day 0 to 11 of erythroid differentiation (n = 3 biologically independent experiments). (f) Colony-forming assay showing numbers of colonies produced by CB-derived $CD34^+$ HSPCs. $CD34^+$ cells were pre-incubated with 10 μM VTX2337 or 10 μM CUCPT8m in expansion medium for 3 days. Then 200 cells were seeded in semi-solid medium containing 10 μM VTX2337 or 10 μM CUCPT8m (n = 3 biologically independent experiments). (g) Representative images of BFU-E from biological triplicate; scale bar = 100 μm. The histogram indicates the area of BFU-E colonies. (h) TLR8 expression in erythroid cells derived from CB $CD34^+$ cells at indicated time points during erythroid differentiation after VTX2337 or CUCPT8m treatment (n = 3 biologically independent experiments). (i) Apoptosis of CFU-E in $CD34^+$ cells on day 6 of erythroid differentiation. CFU-E were gated as $CD123^-CD235a^-CD34^-CD36^+$ followed by apoptosis assay (n = 3 biologically independent experiments). Data are mean ± SEM; statistical significance was determined using the unpaired two-tailed Student's t-test for two groups comparison or two-way ANOVA for multiple comparisons. *$P < 0.05$, **$P < 0.01$, ***$P < 0.001$, ****$P < 0.0001$, ns = not significant.

Supplementary Figure 4

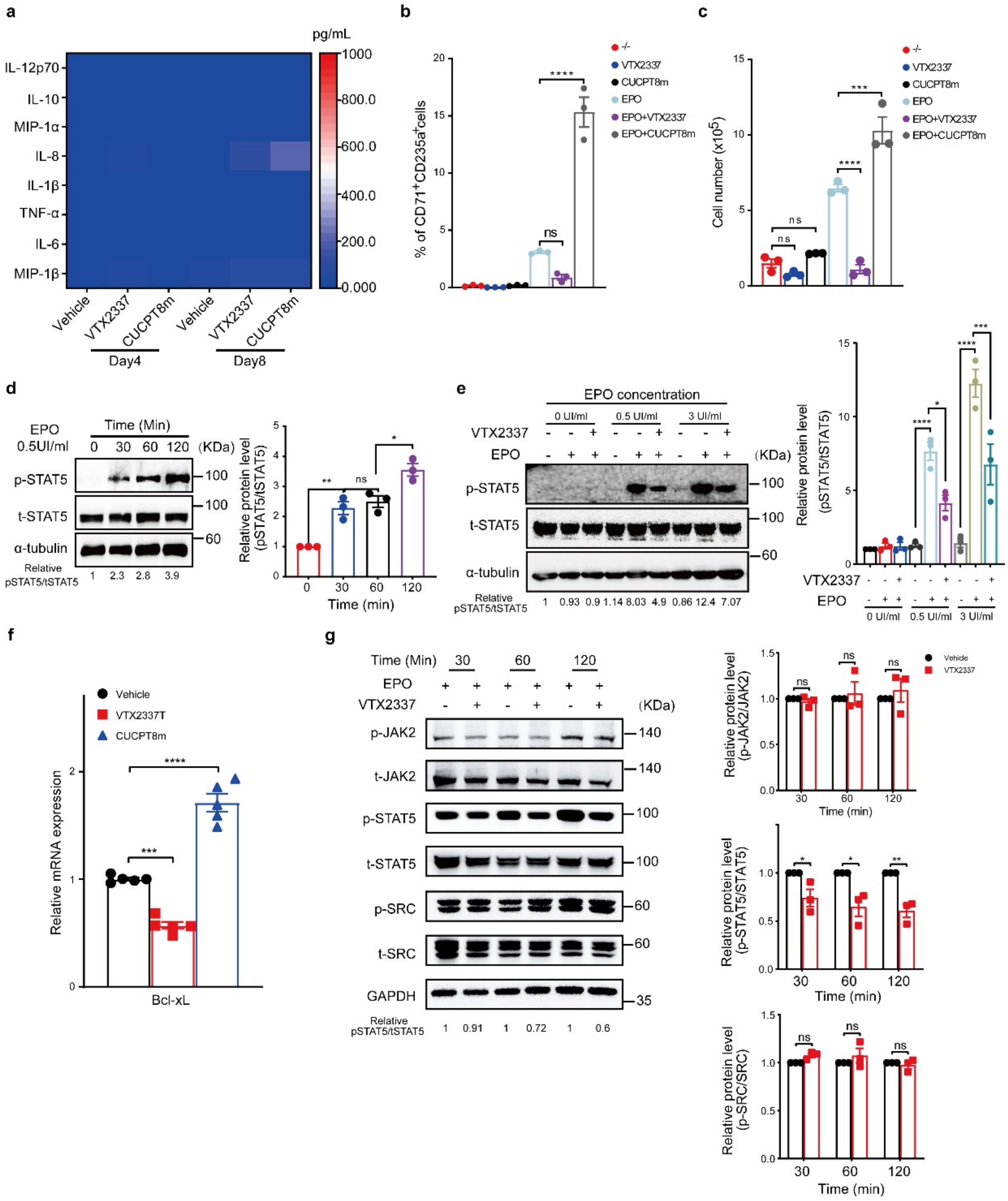

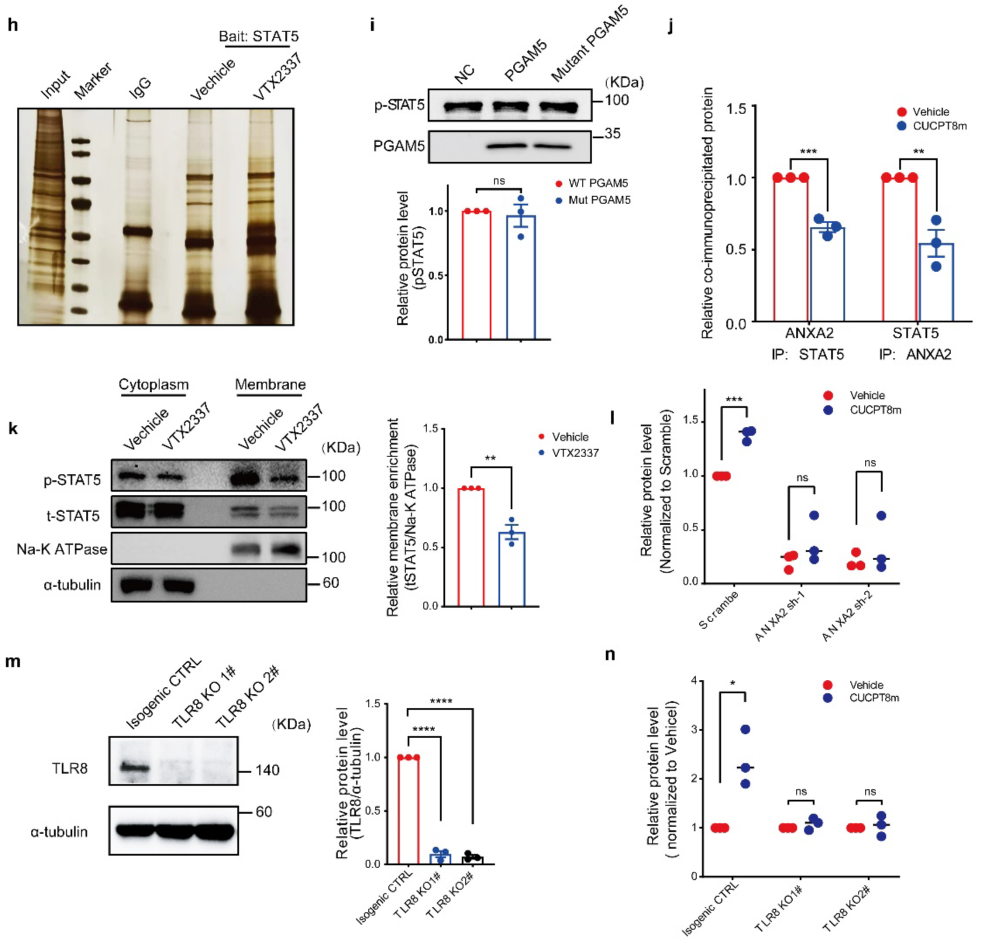

Supplementary Figure 4. Linkage of TLR8 activity with EPO/EPOR/JAK/STAT5 signaling pathways. (a) Cytokine assay of TLR8 downstream targets in the CM (n = 3 biologically independent experiments). (b) The percentage of CD71+CD235a+ cells and cell number (c) from day 0 to day 8 of erythroid differentiation (CB-derived CD34+ cells) (n = 3 biologically independent experiments). (d) Immunoblotting of STAT5 phosphorylation in HuDEP2 cells treated with 0.5 U/ml EPO for various times and the quantitation (n = 3 biologically independent experiments). (e) Immunoblotting of STAT5 phosphorylation in HuDEP2 cells treated with EPO and VTX2337 for 2h and the quantitation (n = 3 biologically independent experiments). (f) Relative expression of *BCL-xL* in day 11-differentiated erythroid cells following treatments (n = 5 biologically independent experiments). (g) Immunoblotting of the indicated proteins in HuDEP2 cells treated with EPO or in combination with VTX2337 for various times and the quantitation (n = 3 biologically independent experiments). (h) Silver staining showing proteins immunoprecipitated by anti-STAT5 antibody from HuDEP2 cells treated with/without 10 μM VTX2337 for 2 h. (i) In vitro phosphatase assay showing the

effects of WT and mutant PGAM5 on p-STAT5 and the quantitation (n = 3 biologically independent experiments). (j) The quantitation of the co-immunoprecipitated ANXA2 and STAT5 with STAT5 and ANAX2 in HuDEP2 cells treated with VTX2337 (n = 3 biologically independent experiments). (k) Immunoblotting of t-STAT5 and p-STAT5 in plasma membrane of HuDEP2 cells treated with vehicle or VTX2337 for 10 minutes and the quantitation. Na-K ATPase is the loading control of membrane protein (n = 3 biologically independent experiments). (l) The quantitation of p-STAT5 in ANXA2-depleted HuDEP2 cells treated with CUCPT8m (n = 3 biologically independent experiments). (m) Immunoblotting of TLR8 in isogenic CTRL and *TLR8*-knockout HuDEP2 cells and the quantitation (n = 3 biologically independent experiments). (n) The quantitation of p-STAT5 in *TLR8*-knockout HuDEP2 cells treated with CUCPT8m (n = 3 biologically independent experiments). Data are mean ± SEM; statistical significance was determined using the unpaired two-tailed Student's t-test for two groups comparison or one-way ANOVA for multiple comparisons. **P* < 0.05, ***P* < 0.01, ****P* < 0.001, *****P* < 0.0001, ns = not significant.

Supplementary Figure 5

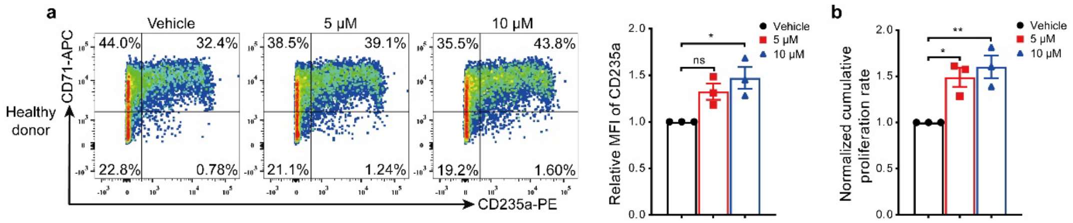

Supplementary Figure 5. TLR8 inhibition improves erythropoiesis of CD34⁺ cells from adult BM. (a) Representative flow cytometry showing erythroid differentiation of cells from healthy adult BM on day 11 of differentiation and the statistics of the relative MFI of CD235a (n = 3 biologically independent experiments). (b) The statistics of the normalized cumulative proliferation rate (n = 3 biologically independent experiments). Data are mean ± SEM; statistical significance was determined using the unpaired two-tailed Student's t-test. *P < 0.05, **P < 0.01.

Supplementary tables

Supplementary table 1. Patient information of family members suffering from various diseases.

Patients	Diseases	Gender	Life-span
G2-1	Leukemia	Male	20-yr
G2-3	Leukemia	Male	20-yr
G2-5	Infertity	Female	--
G3-2	Leukemia	Female	46-yr
G3-3	Lymphoma	Male	50-yr
G3-10	Pure red cell aplasia	Female	--
G4-4	Rheumatism	Female	--
G4-15	Anemia	Male	7-month
G5-4(Proband)	Inherited non-hemolytic anemia	Male	4-yr

Supplementary table 2. Sequences of gRNA, qPCR primers, and shRNA used.

name	sequences
sgRNA-1	TGCCGGGTATCTTTTACCAACGG
sgRNA-2	GCTTAGACTCATTATGGTGGTGG
sgRNA-3	ATACTGGATAGAGGATGCTATGG
sgRNA-4	CACCGTTGGTAAAAGATACC
BCL-xL: Forward	5’-AAGAGAACAGGACTGAGGCC-3’
BCL-xL: Reverse	5’-TTGCTTTACTGCTGCCATGG-3’
18S: Forward	5’-ACCGCAGCTAGGAATAATGGA-3’
18S: Reverse	5’-GCCTCAGTTCCGAAAACCA-3’
ANXA2 shRNA 1#	5’-GCAGGAAATTAACAGAGTCTA-3’
ANXA2 shRNA 2#	5’-CGGGATGCTTTGAACATTGAA-3’

Raw data of Western blotting

Figure 3c

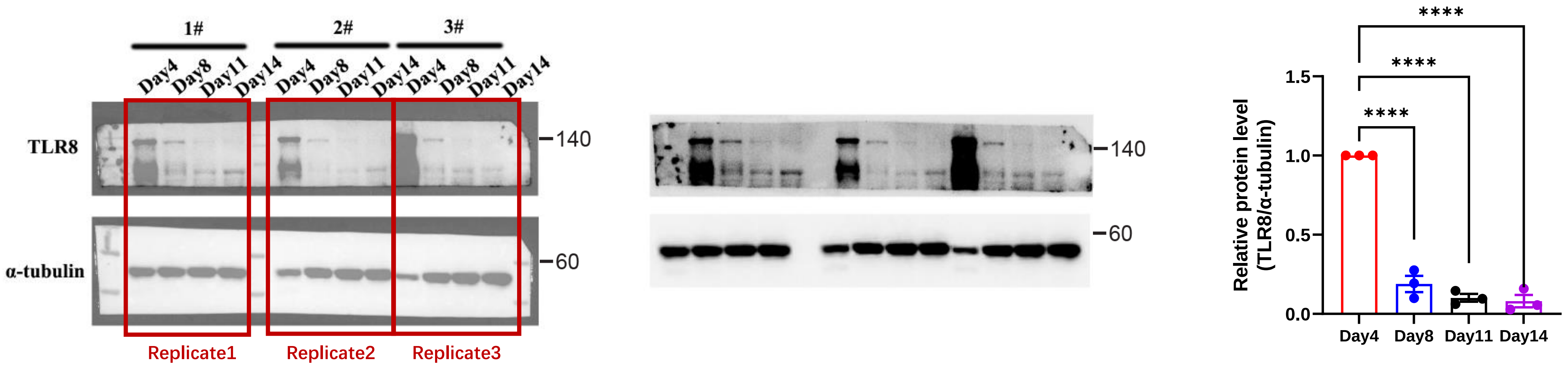

Day4			Day8			Day11			Day14		
1	1	1	0.275402288	0.192965949	0.099754256	0.145630666	0.096210361	0.061639043	0.159358356	0.056072749	0.027823028

anti-TLR8 (CAT# 11886, CST)
anti-α-tubulin (CAT#ab11304, Abcam)

Figure 3f

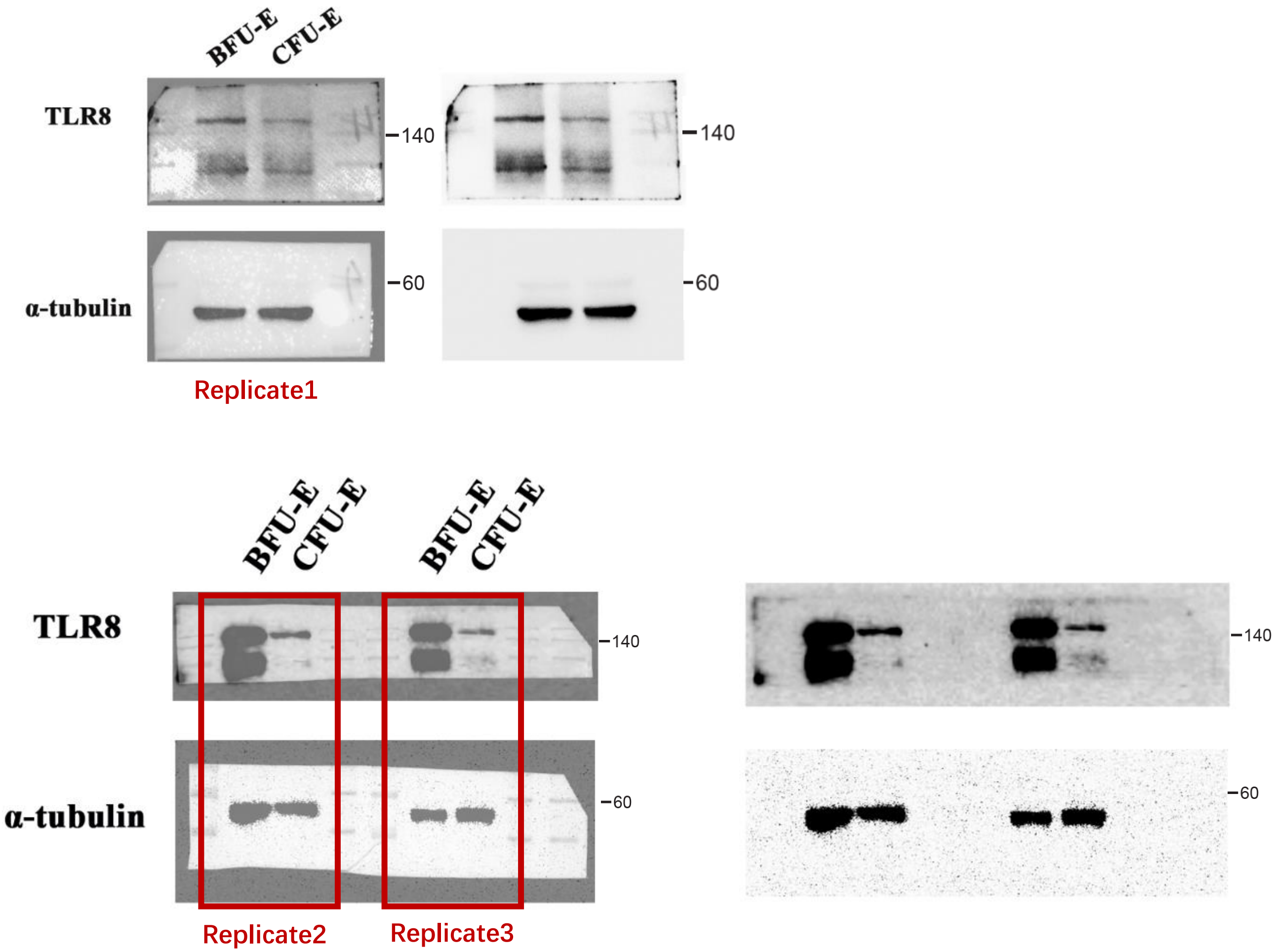

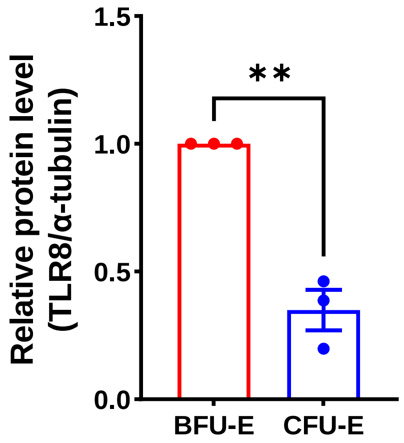

BFU-E			CFU-E		
1	1	1	0.461869398	0.38716814	0.198076775

anti-TLR8 (CAT# 11886, CST)
anti-α-tubulin (CAT#ab11304, Abcam)

Figure 6b

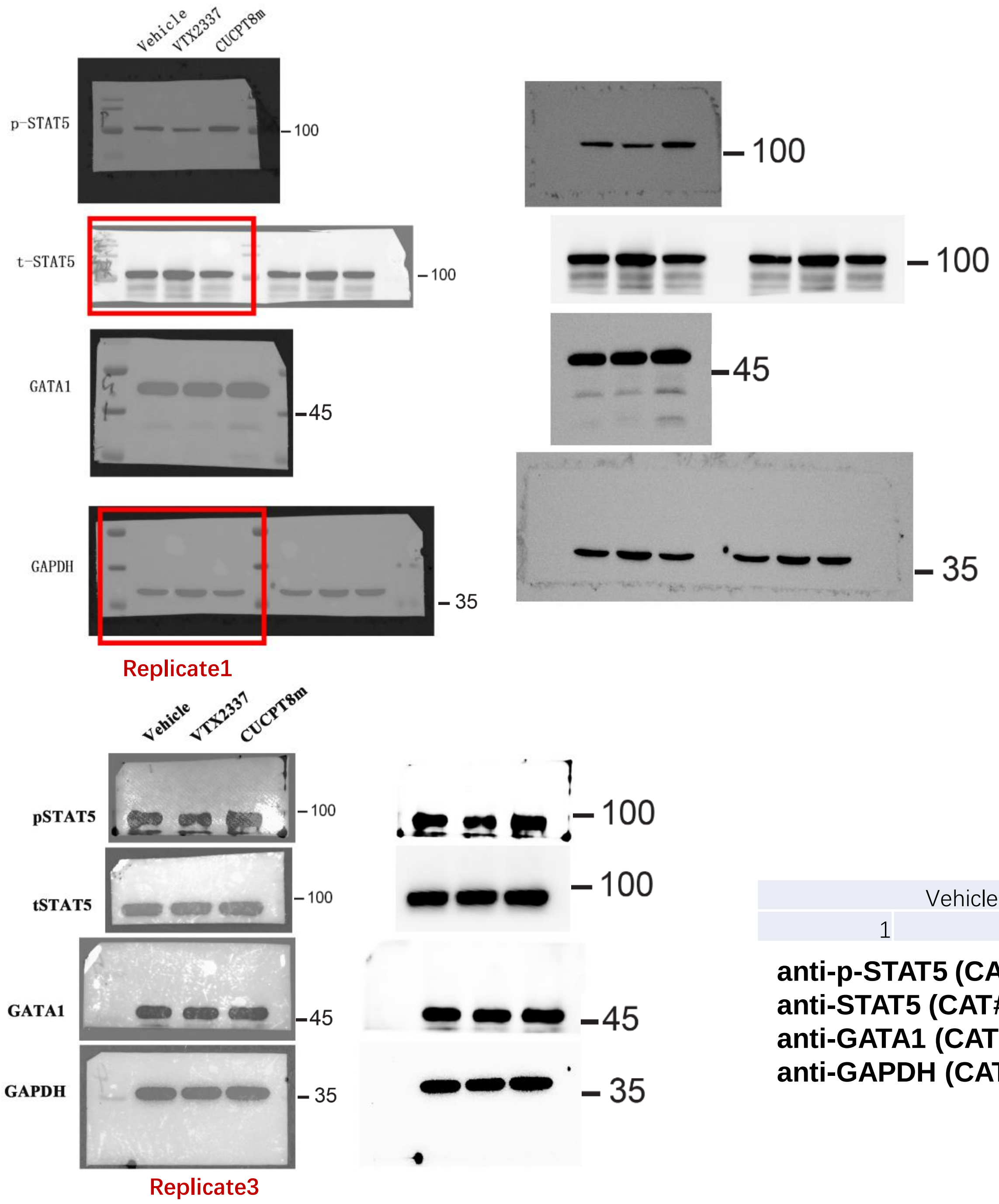

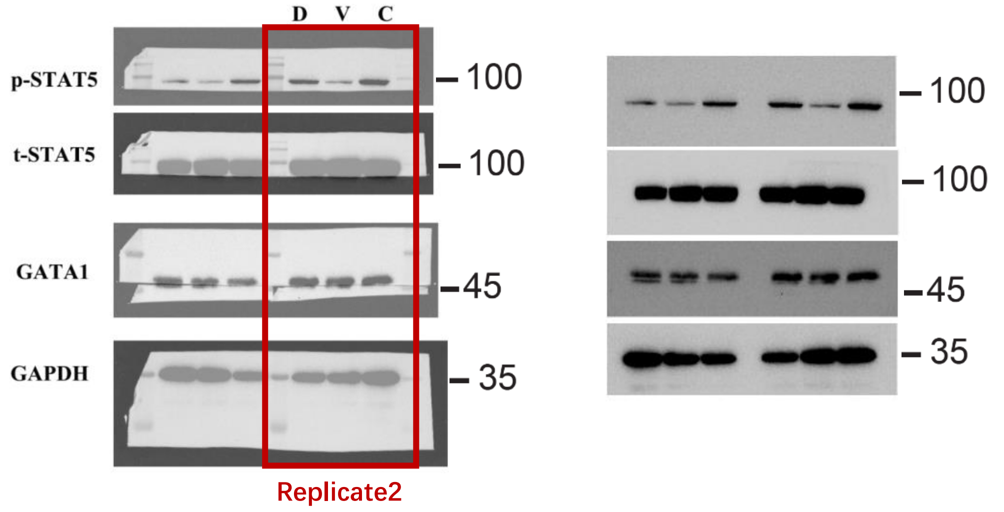

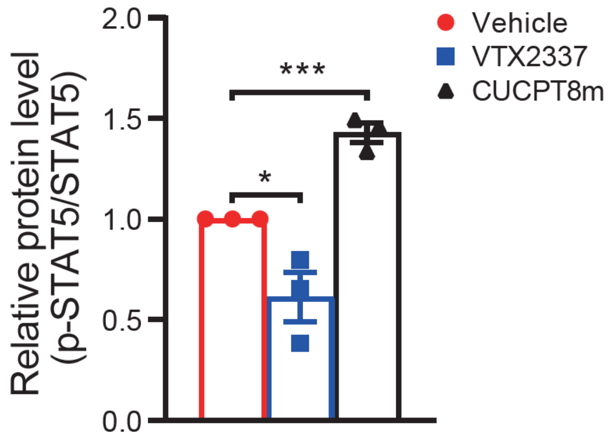

Vehicle			VTX2337			CUCPT8m		
1	1	1	0.654740885	0.382420445	0.798265088	1.456249317	1.494917529	1.336115384

anti-p-STAT5 (CAT# 9359, CST)
anti-STAT5 (CAT# 94205, CST)
anti-GATA1 (CAT#ab181544, Abcam)
anti-GAPDH (CAT# 60004-1-Ig, Proteintech)

Figure 6c

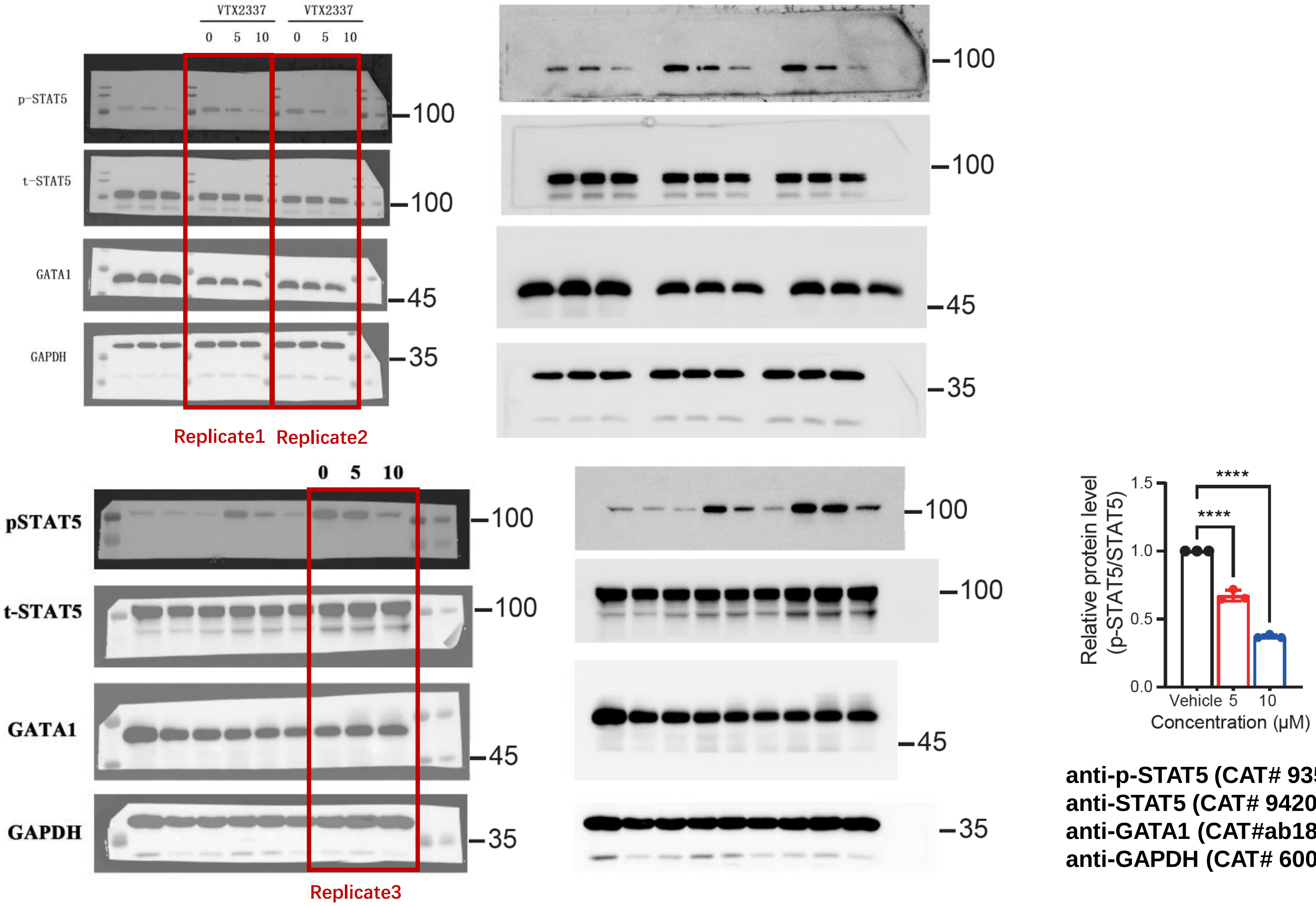

anti-p-STAT5 (CAT# 9359, CST)
anti-STAT5 (CAT# 94205, CST)
anti-GATA1 (CAT#ab181544, Abcam)
anti-GAPDH (CAT# 60004-1-Ig, Proteintech)

Vehicle			VTX2337			CUCPT8m		
1	1	1	0.645615382	0.652577857	0.719593252	0.387539504	0.364896339	0.364147773

Figure 6d

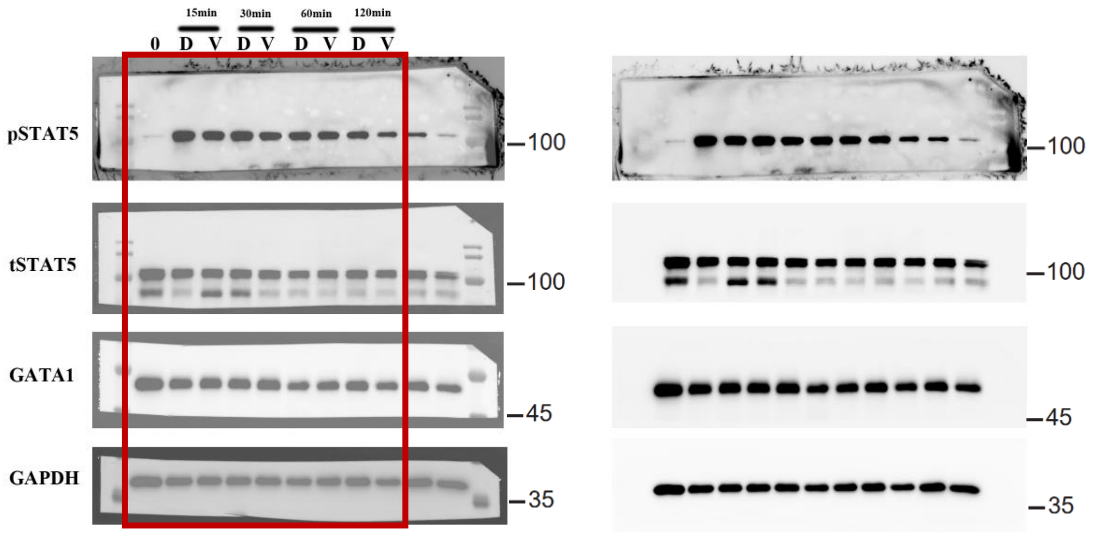

Replicate1

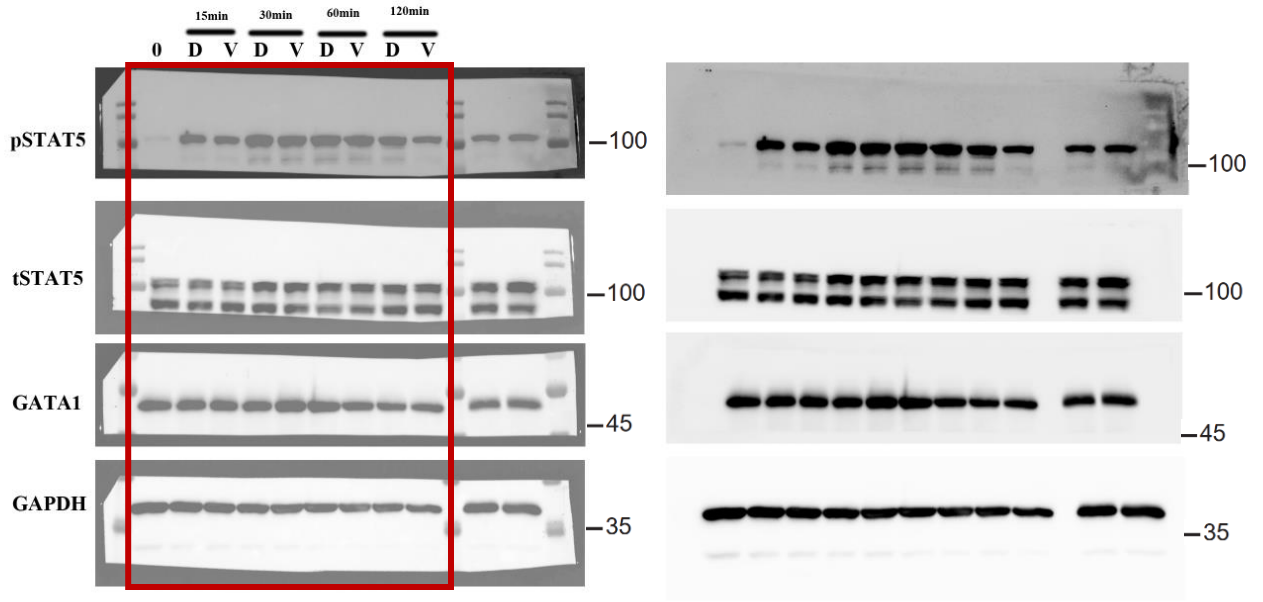

Replicate2

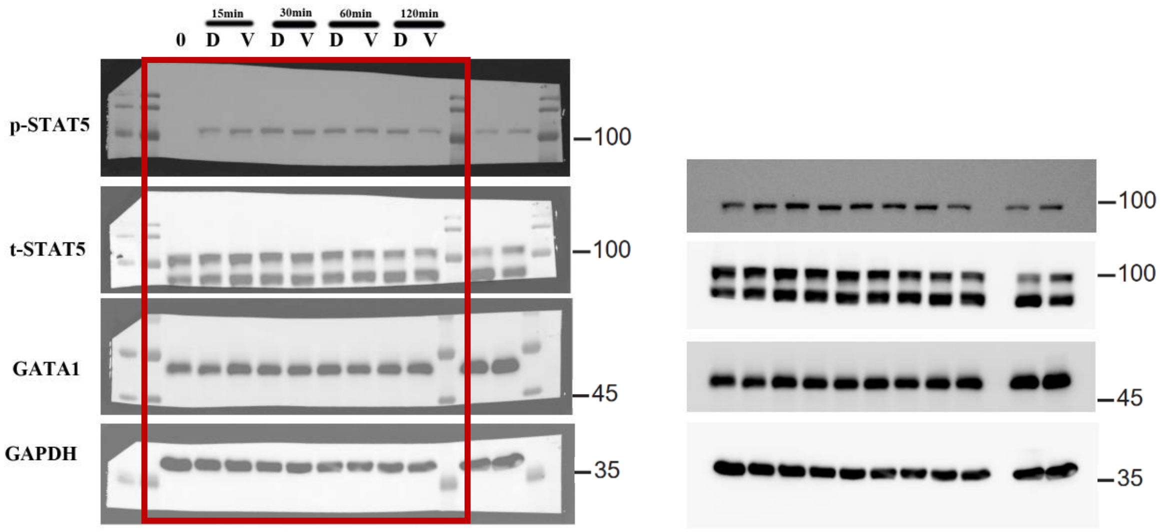

Replicate3

15min	Vehicle	1	1	1
	VTX2337	1.055886998	0.615346632	0.687544014
30min	Vehicle	1	1	1
	VTX2337	0.91954023	2.625	0.990951624
60min	Vehicle	1	1	1
	VTX2337	0.886075949	0.940043492	0.736010533
120min	Vehicle	1	1	1
	VTX2337	0.587755102	0.407930059	0.330457863

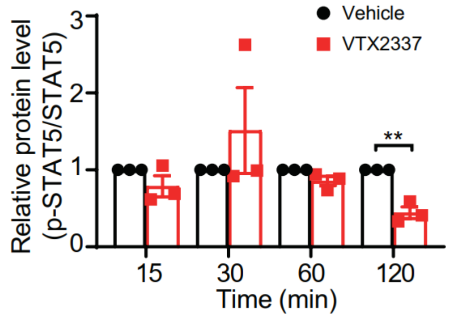

anti-p-STAT5 (CAT# 9359, CST)
anti-STAT5 (CAT# 94205, CST)
anti-GATA1 (CAT#ab181544, Abcam)
anti-GAPDH (CAT# 60004-1-Ig, Proteintech

Figure 6e

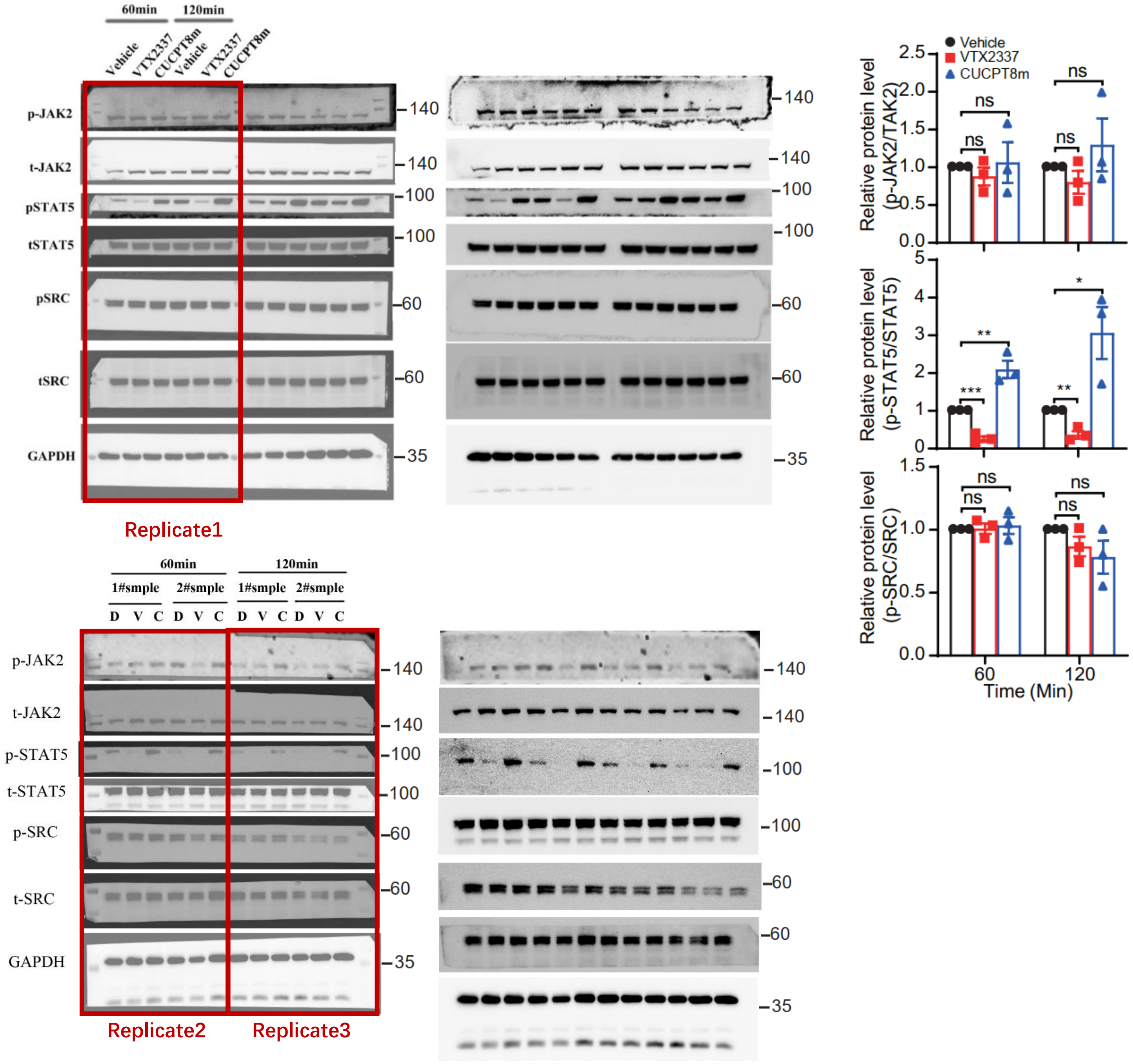

P-JAK2	60min	Vehicle	1	1	1
		VTX2337	0.662190559	0.89630643	1.075630252
		CUCPT8m	0.657172785	0.95904691	1.572286057
	120min	Vehicle	1	1	1
		VTX2337	1.070588235	0.787780656	0.544811321
		CUCPT8m	1.062178828	1.983870968	0.84258269
P-STAT5	60min	Vehicle	1	1	1
		VTX2337	0.382766934	0.231320767	0.140588989
		CUCPT8m	2.545027167	1.952374316	1.788457244
	120min	Vehicle	1	1	1
		VTX2337	0.322216198	0.551276182	0.217290855
		CUCPT8m	1.699208688	3.927733416	3.559463157
P-SRC	60min	Vehicle	1	1	1
		VTX2337	1.067943773	0.928325384	1.029938272
		CUCPT8m	1.143805572	0.912571358	1.044444444
	120min	Vehicle	1	1	1
		VTX2337	1.008727273	0.856609874	0.736914912
		CUCPT8m	1.00102981	0.548037462	0.797474054

anti-p-STAT5 (CAT# 9359, CST)
anti-STAT5 (CAT# 94205, CST)
anti-JAK2 (CAT# 3230, CST)
anti-p-JAK2 （CAT# 3771, CST)
anti-SRC (CAT#110971-AP, Proteintech)
anti-p-SRC (CAT# 6943, CST)
anti-GAPDH (CAT# 60004-1-Ig, Proteintech)

Figure 6g

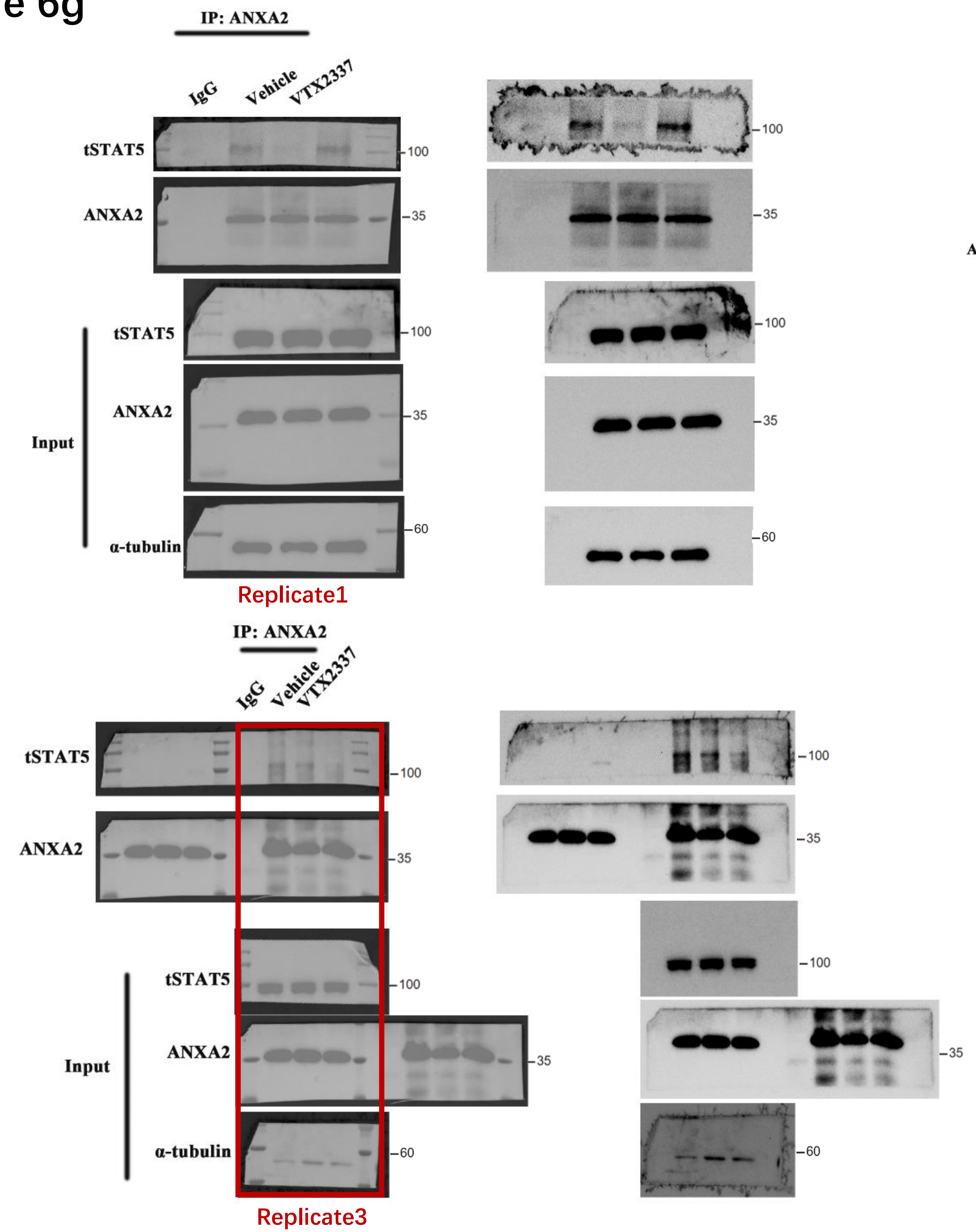

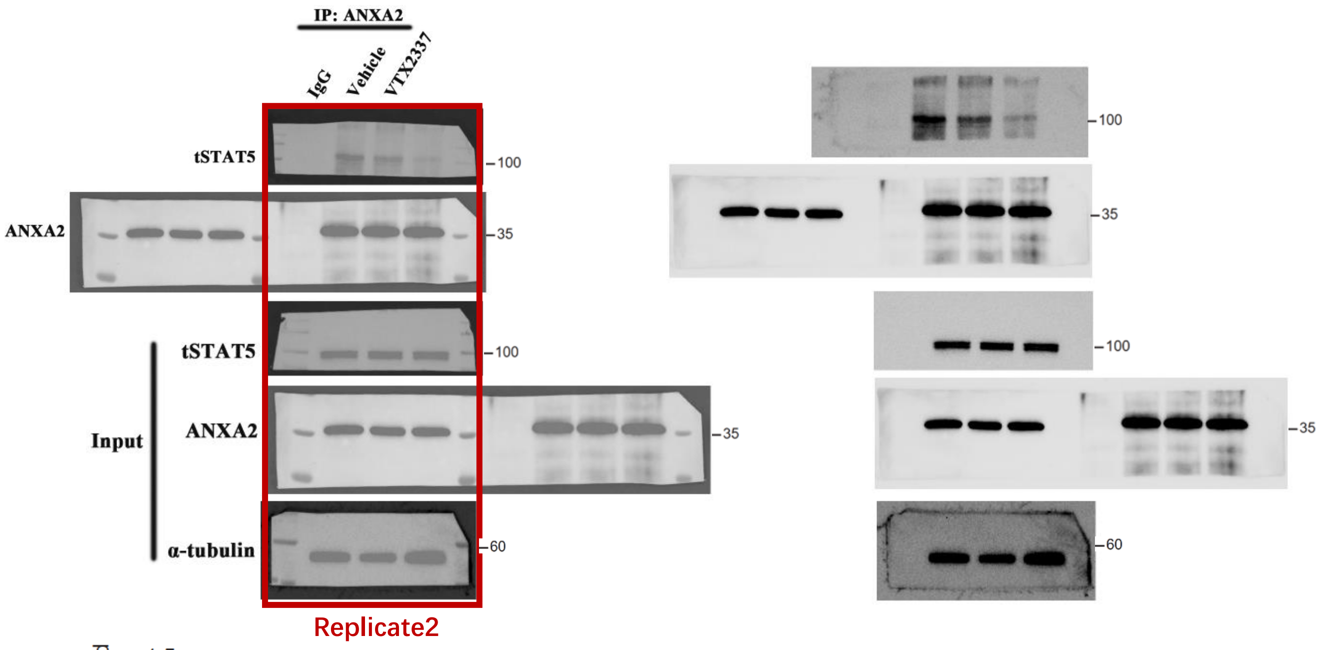

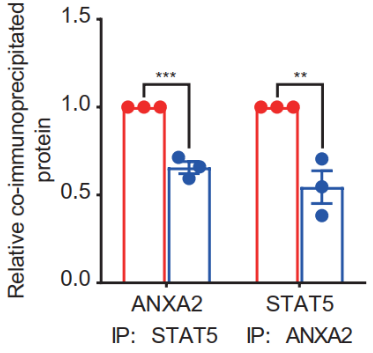

	Vehicle	VTX2337			Vehicle	VTX2337
CoIP: STAT5	1	0.594984432		CoIP: ANXA2	1	0.38366583
	1	0.71400208			1	0.546665362
	1	0.661117451			1	0.705978719

anti-STAT5 (CAT# 94205, CST)
anti-ANXA2(CAT# 11256-1-AP, Proteintech)
anti-α-tubulin (CAT#ab11304, Abcam)

Figure 6g

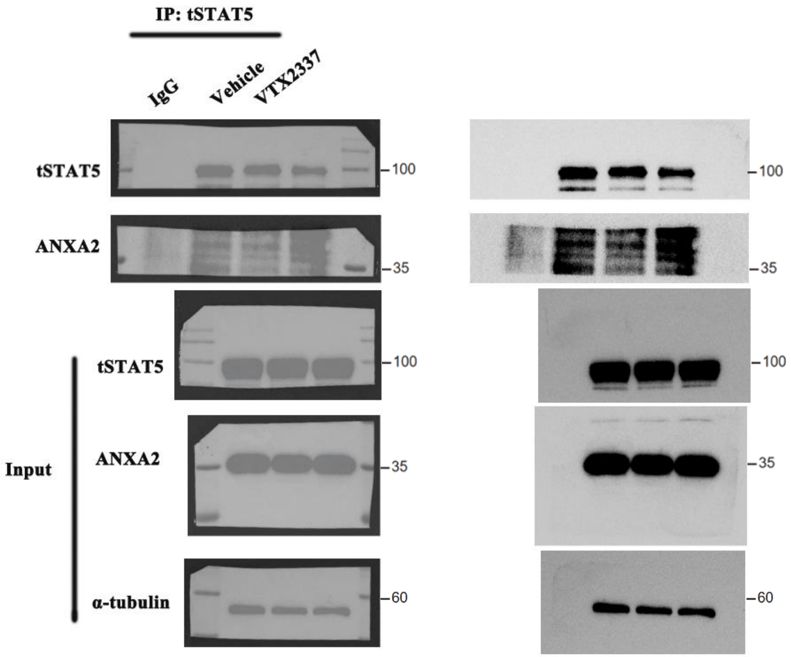

Replicate1

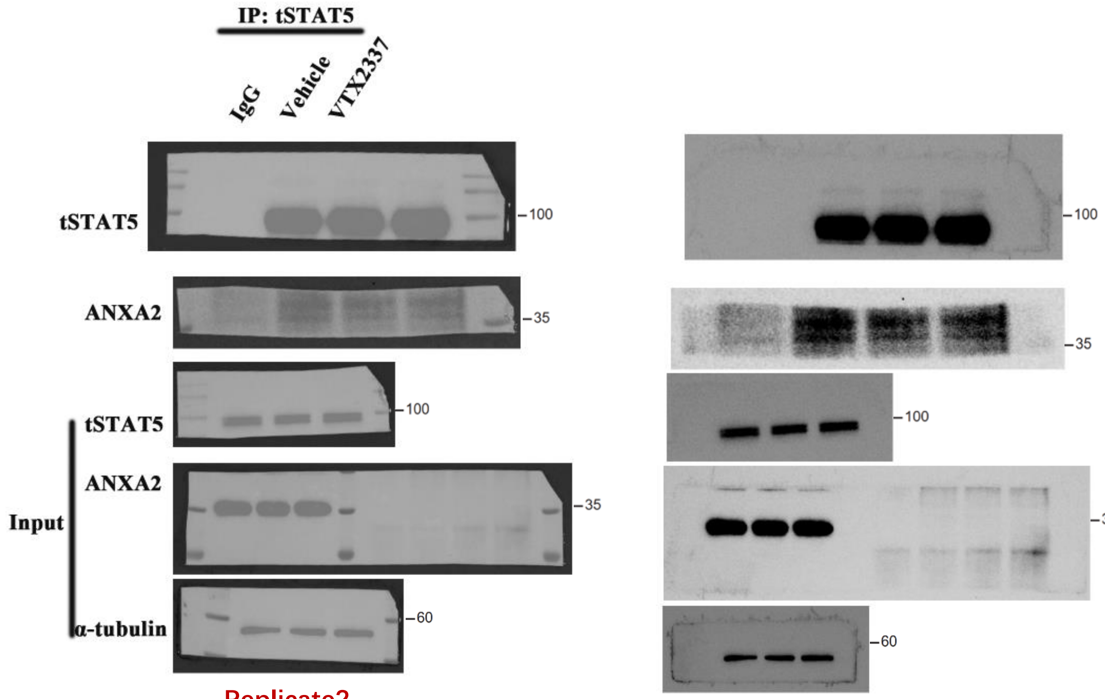

Replicate2

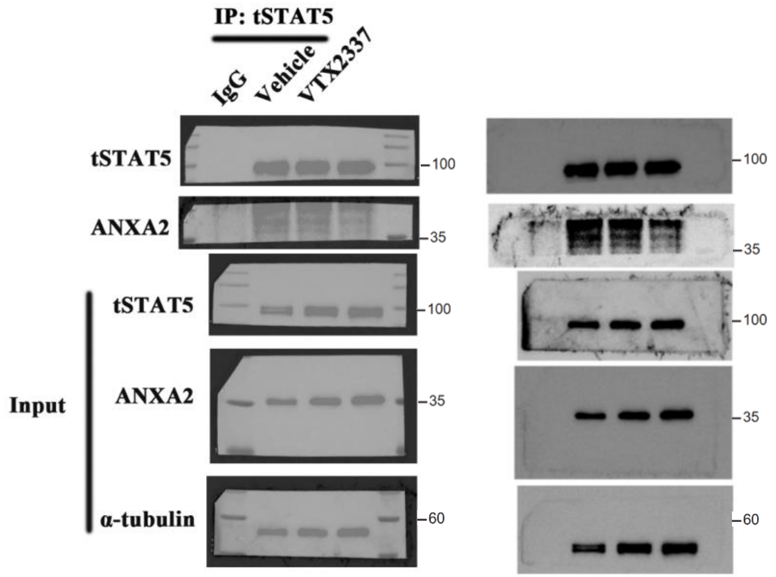

Replicate3

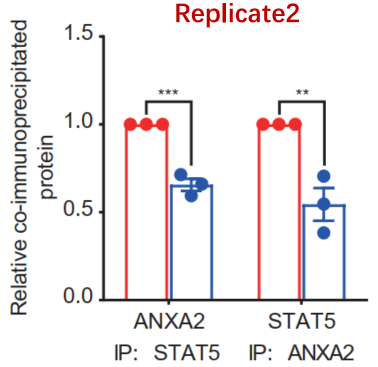

	Vehicle	VTX2337			Vehicle	VTX2337
CoIP: STAT5	1	0.594984432		CoIP: ANXA2	1	0.38366583
	1	0.71400208			1	0.546665362
	1	0.661117451			1	0.705978719

anti-STAT5 (CAT# 94205, CST)
anti-ANXA2(CAT# 11256-1-AP, Proteintech)
anti-α-tubulin (CAT#ab11304, Abcam)

Figure 6j

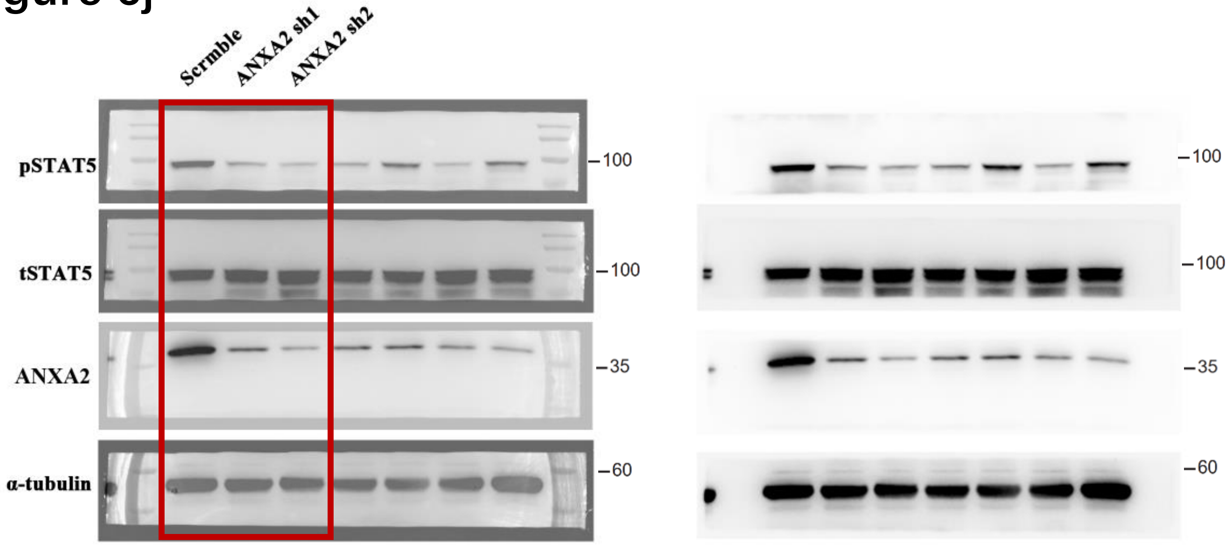

Replicate1

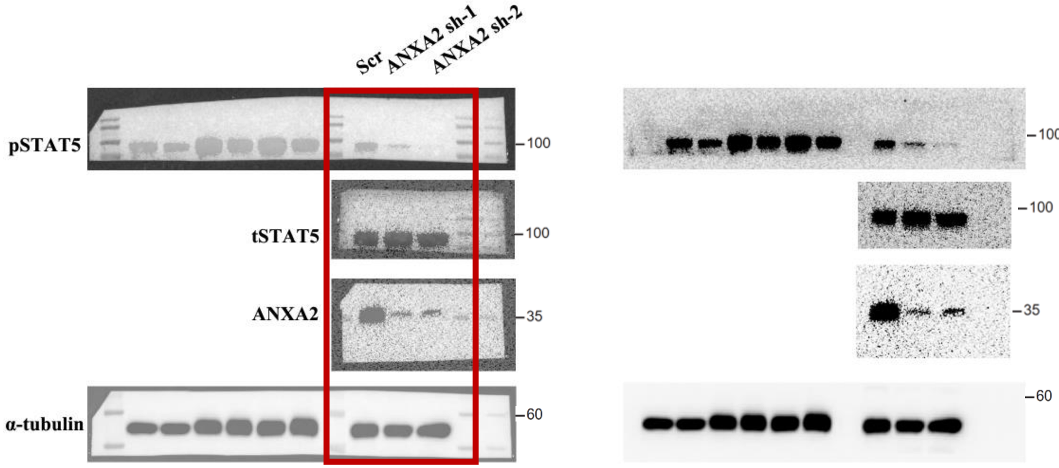

Replicate2

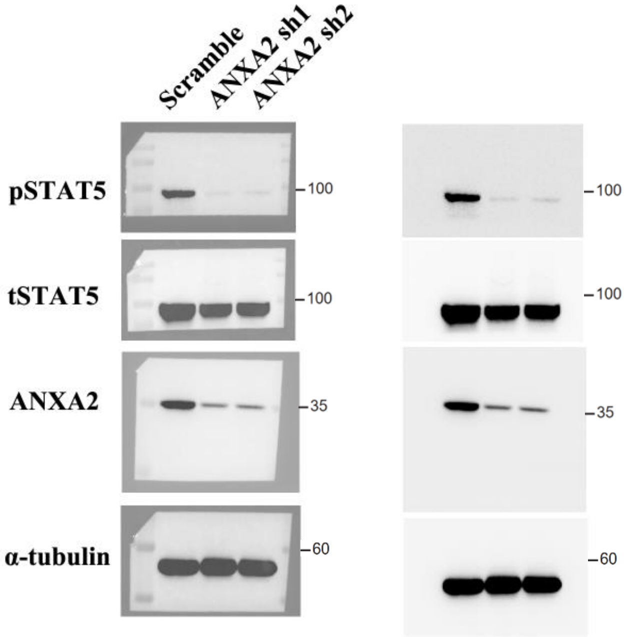

Replicate3

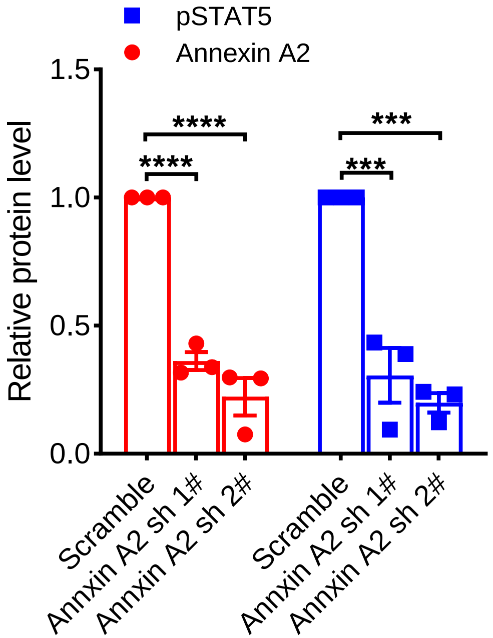

	pSTAT5			Annexin A2		
Scramble	1	1	1	1	1	1
Annxin A2 sh 1#	0.389256762	0.43436	0.094802038	0.338283814	0.430546	0.316629424
Annxin A2 sh 2#	0.231596877	0.241498	0.123507063	0.075236872	0.294629	0.297656052

anti-p-STAT5 (CAT# 9359, CST)
anti-STAT5 (CAT# 94205, CST)
anti-ANXA2(CAT# 11256-1-AP, Proteintech)
anti-α-tubulin (CAT#ab11304, Abcam)

Figure 6k

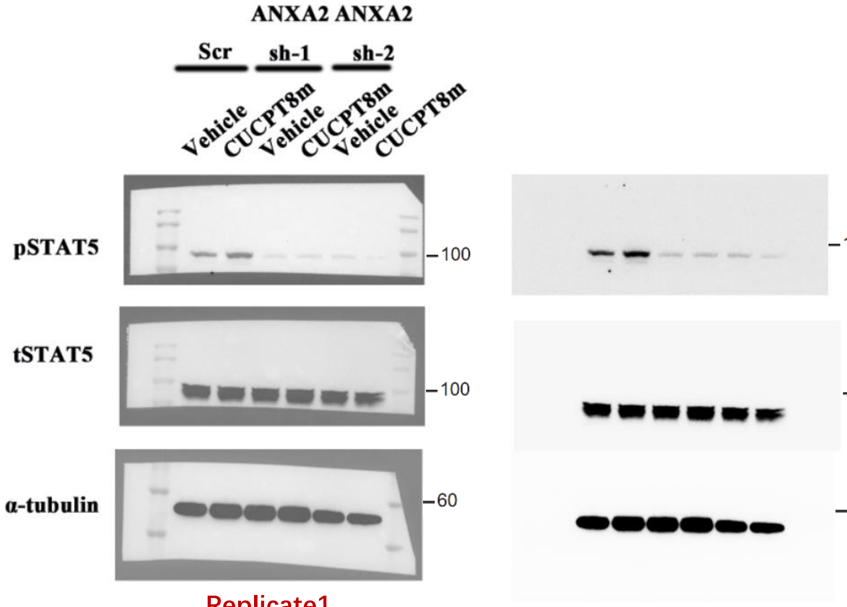

Replicate1

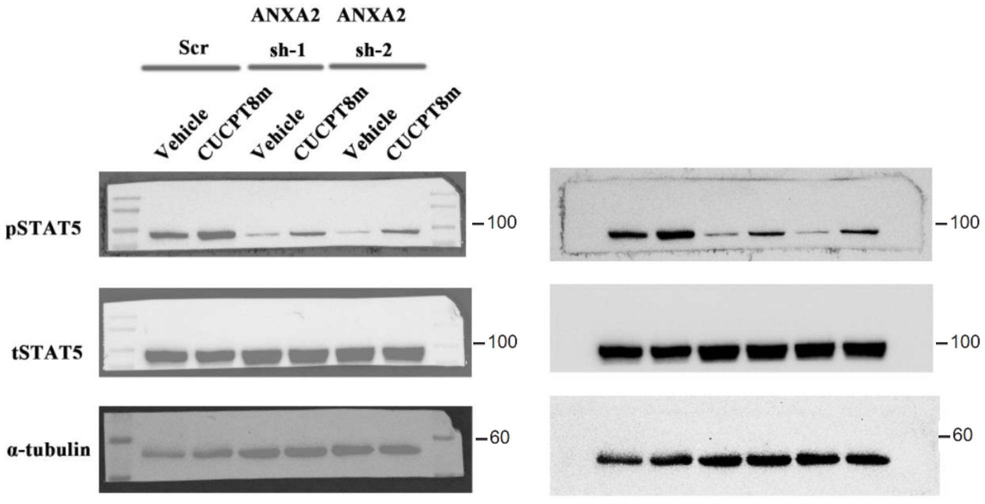

Replicate2

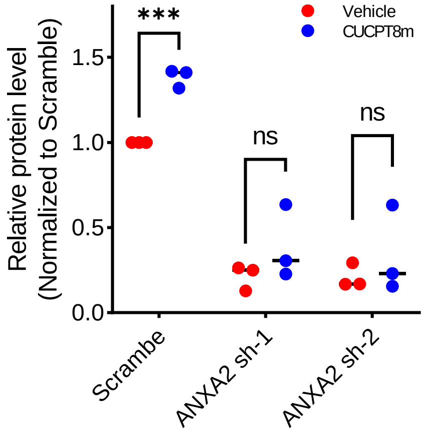

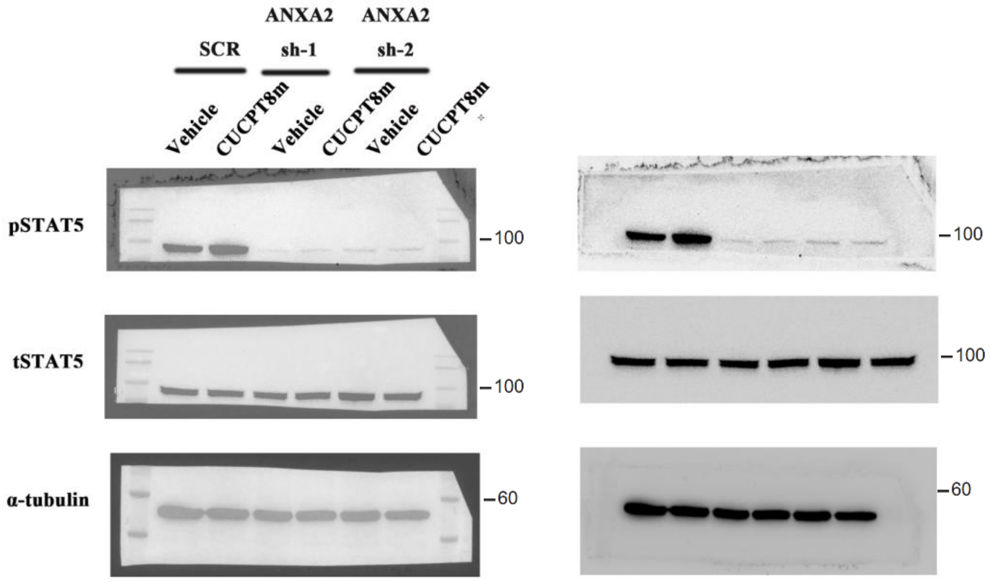

Replicate3

	Vehicle			CUCPT8m		
Scrambe	1	1	1	1.411346569	1.473205805	1.225458682
ANXA2 sh-1	0.249535069	0.263688498	0.638037477	0.30598999	0.634544434	0.81050737
ANXA2 sh-2	0.293733117	0.168595955	0.42539125	0.155404067	0.632376134	0.818889642

anti-p-STAT5 (CAT# 9359, CST)
anti-STAT5 (CAT# 94205, CST)
anti-ANXA2(CAT# 11256-1-AP, Proteintech)
anti-α-tubulin (CAT#ab11304, Abcam)

Figure 6I

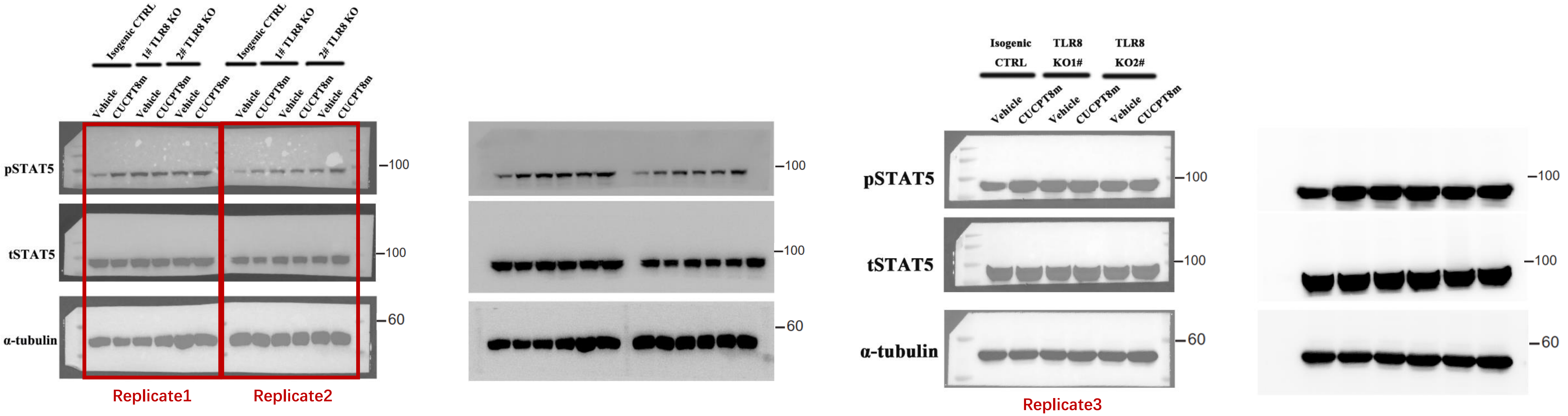

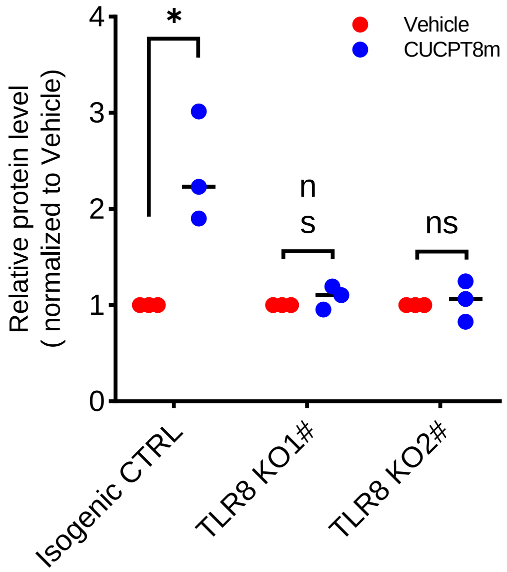

	Vehicle			CUCPT8m		
Isogenic CTRL	1	1	1	2.230121387	3.013529527	1.901307129
TLR8 KO1#	1	1	1	1.195086025	1.105507724	0.954461597
TLR8 KO2#	1	1	1	0.827330097	1.247594776	1.064790326

anti-p-STAT5 (CAT# 9359, CST)
anti-STAT5 (CAT# 94205, CST)
anti-α-tubulin (CAT#ab11304, Abcam)

Figure 7a

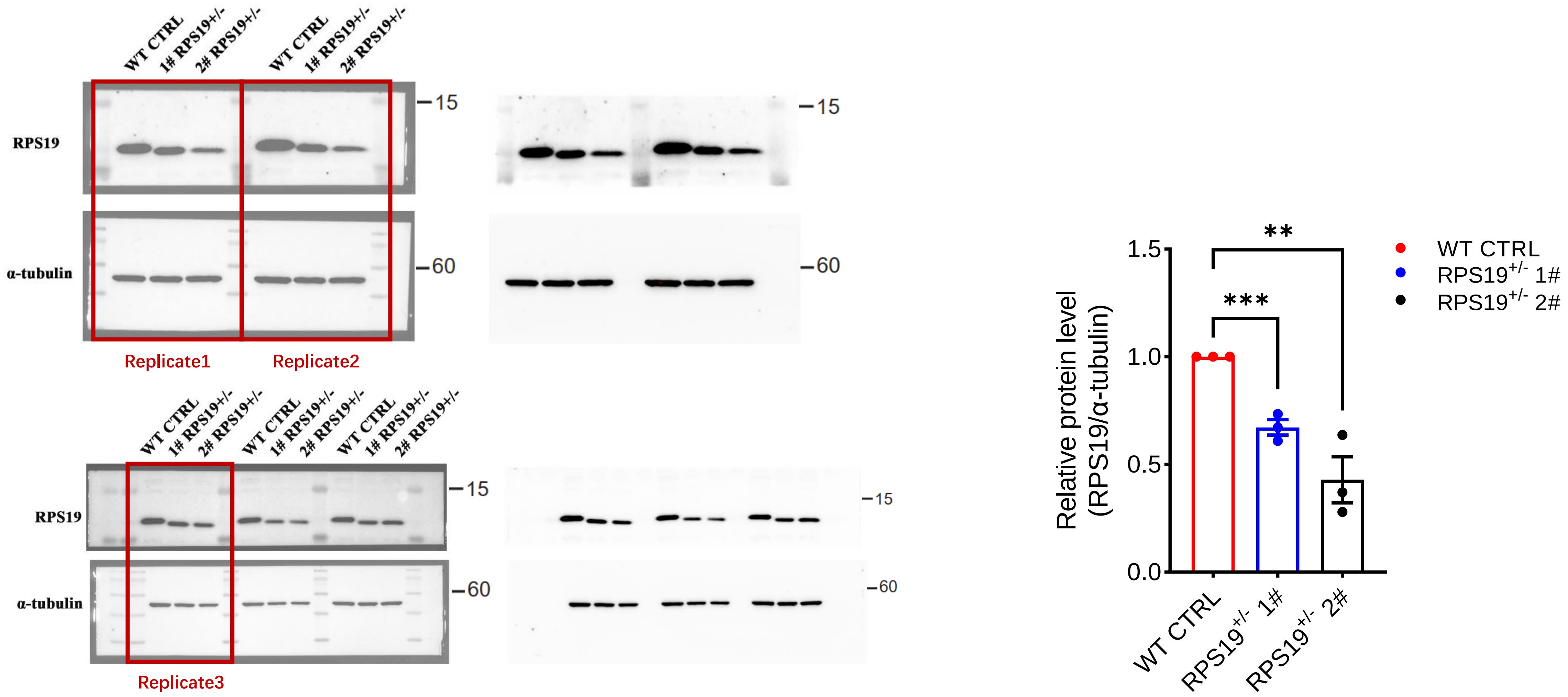

CTRL			RPS19+/- 1#			RPS19+/- 2#		
1	1	1	0.671885387	0.609468	0.734748	0.370211	0.279894	0.636953

anti-RPS19 (CAT# SC-100836, Santa Cruz)
anti-α-tubulin (CAT#ab11304, Abcam)

Figure 7e

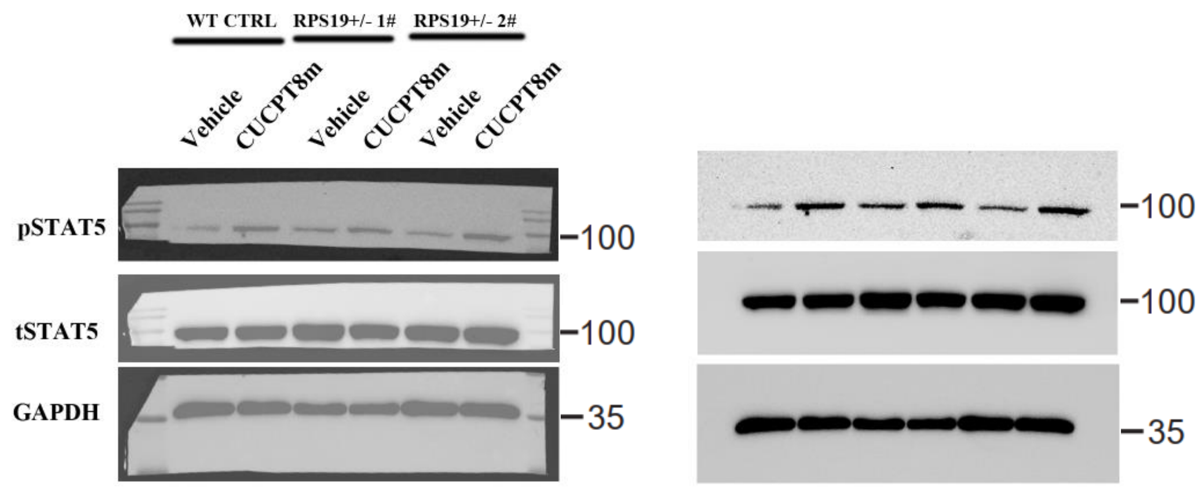

Replicate1

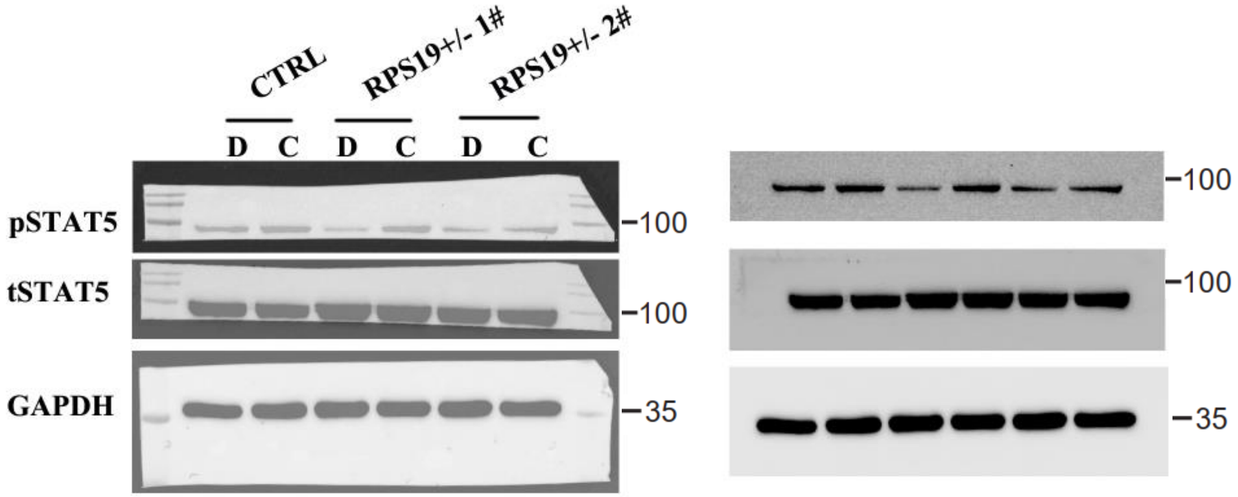

Replicate2

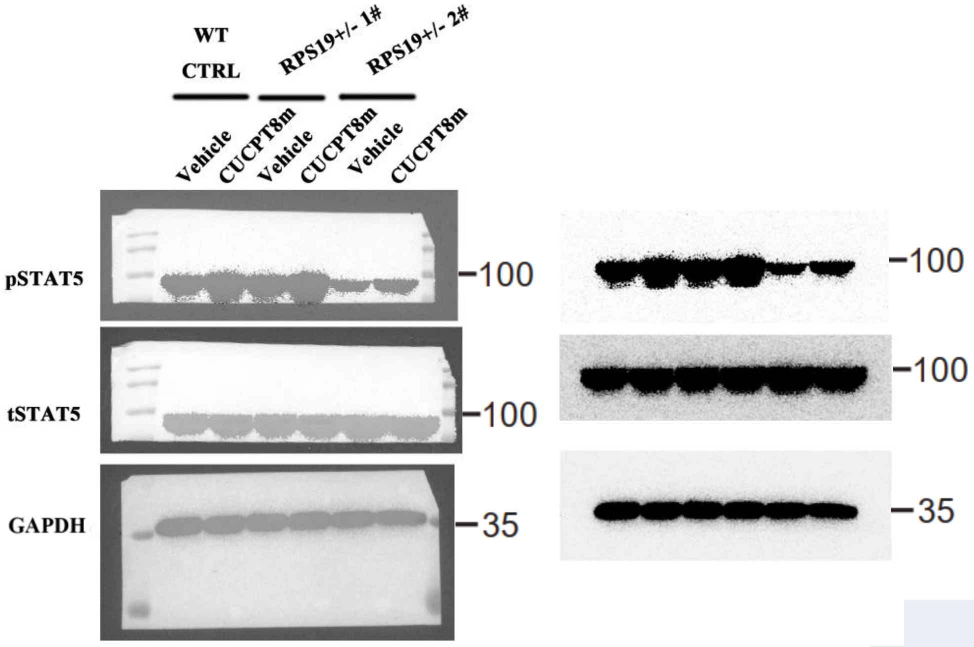

Replicate3

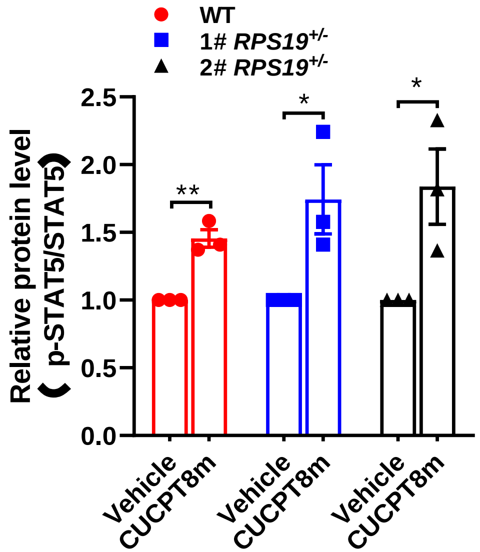

anti-p-STAT5 (CAT# 9359, CST)
anti-STAT5 (CAT# 94205, CST)
anti-α-tubulin (CAT#ab11304, Abcam)

	WT			1# RPS19+/-			2# RPS19+/-		
Vehicle	1	1	1	1	1	1	1	1	1
CUCPT8m	1.583421969	1.410276429	1.371981203	1.577338869	2.24322464	1.409776898	1.818536273	1.365112254	2.329144834

Figure S2a HEK293T OE

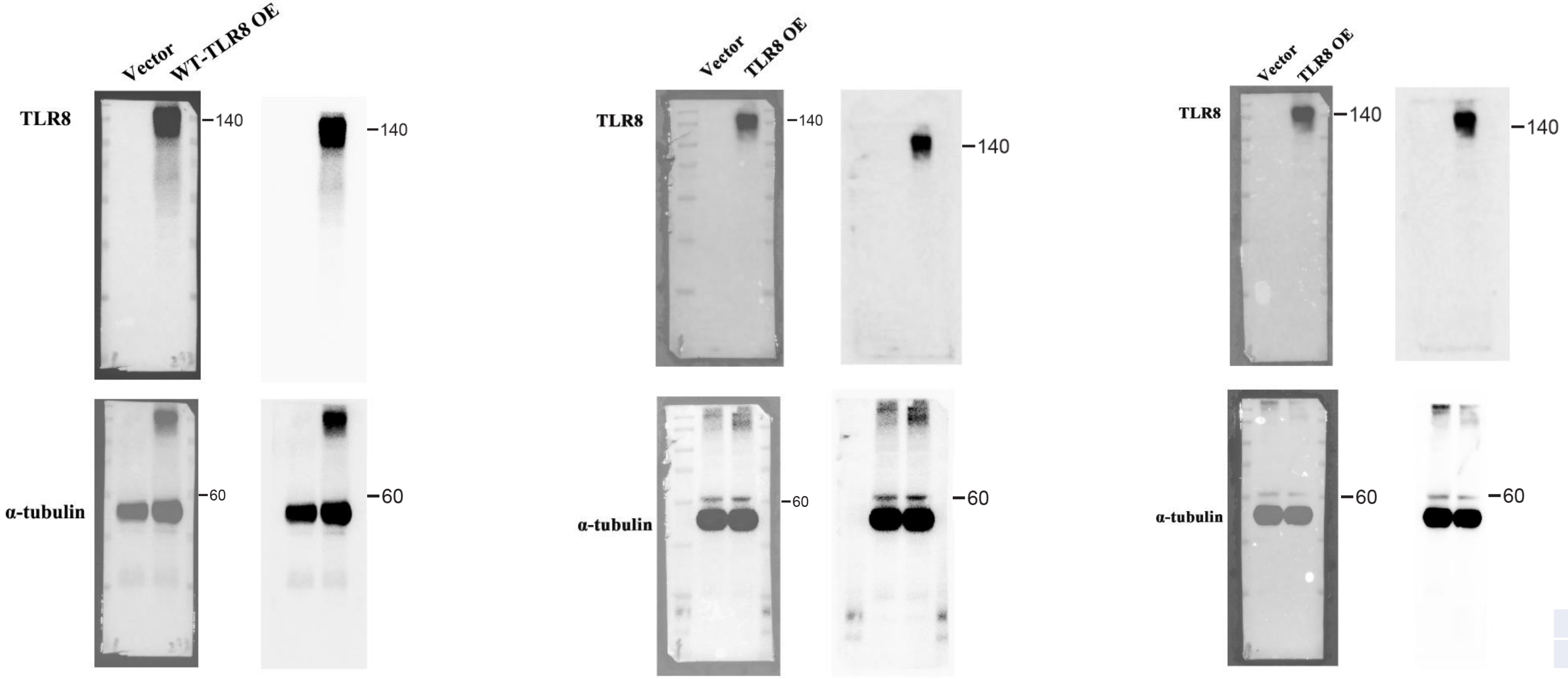

Replicate 1 | Replicate2 | Replicate3

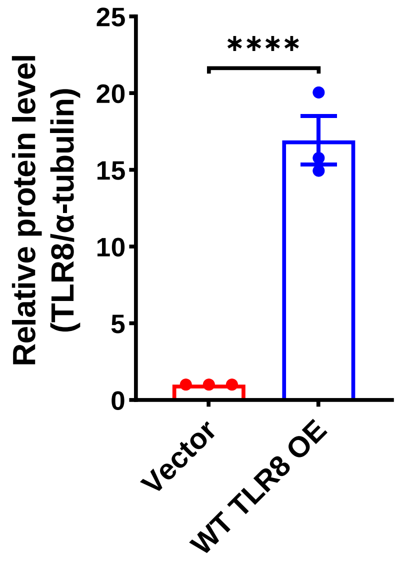

Vector			WT TLR8 OE		
1	1	1	15.7864	14.94094	20.05769

anti-TLR8 (CAT# 11886, CST)
anti-α-tubulin (CAT#ab11304, Abcam)

Figure S2a THP1+IFNγ

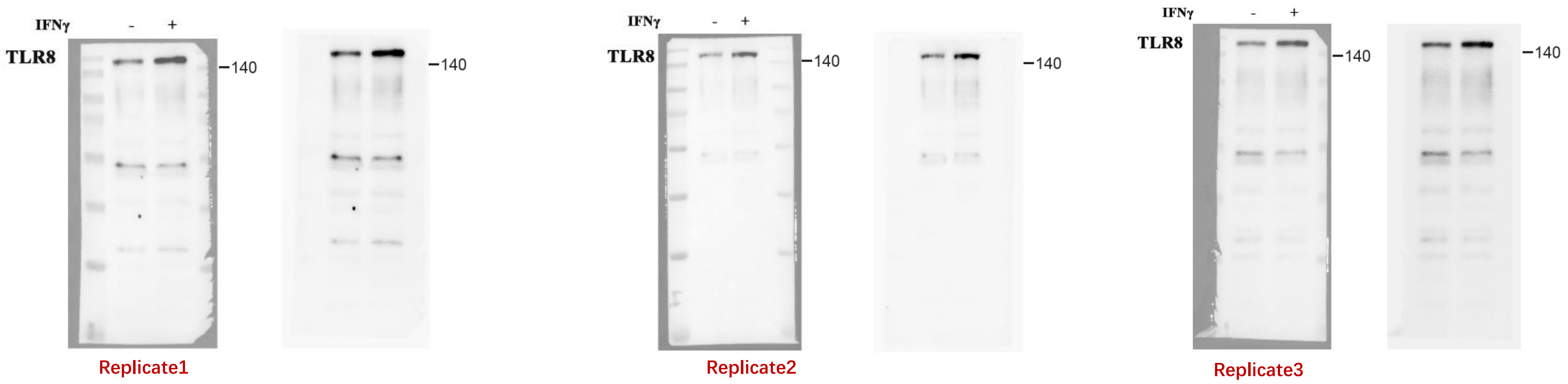

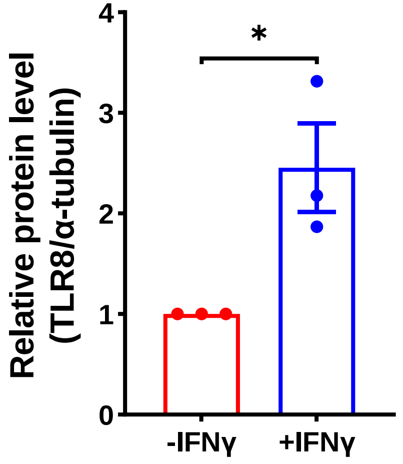

	-IFNγ			+IFNγ	
1	1	1	3.316017	1.869325	2.179708

anti-TLR8 (CAT# 11886, CST)
anti-α-tubulin (CAT#ab11304, Abcam)

Figure S2b

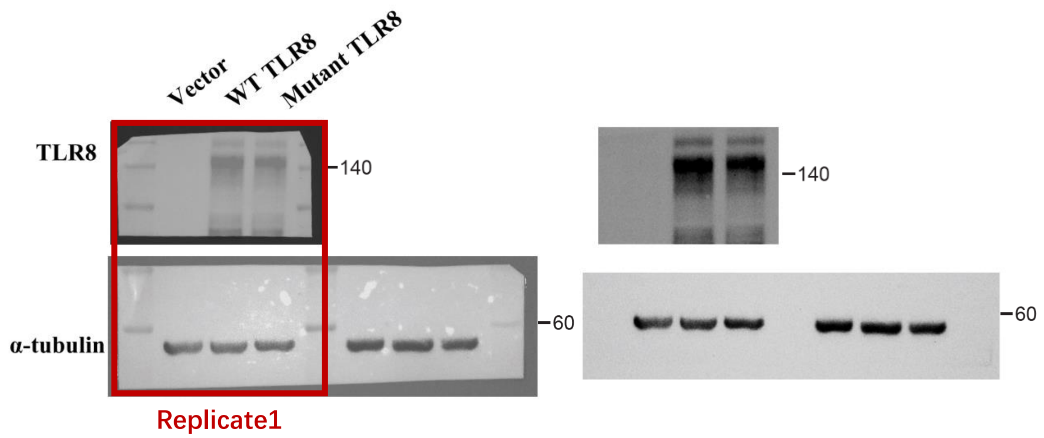

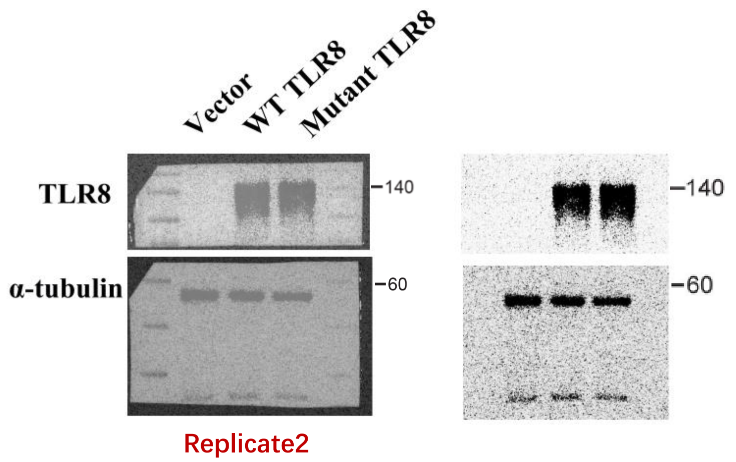

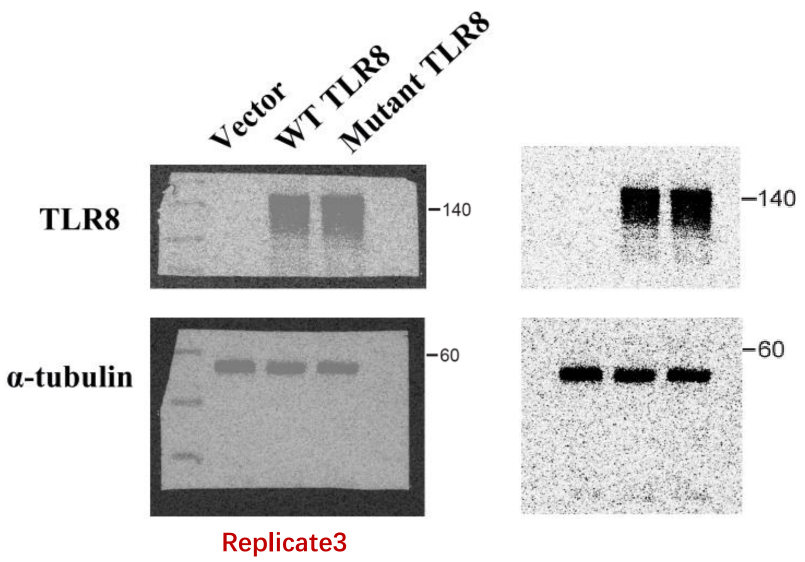

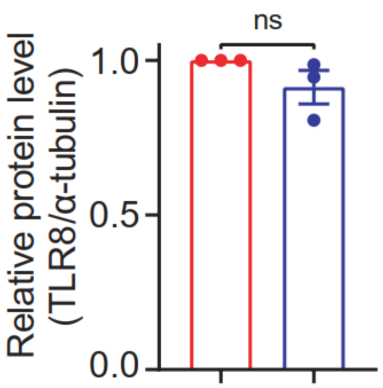

WT			Mut		
1	1	1	0.806642465	0.945566084	0.985754011

anti-TLR8 (CAT# 11886, CST)
anti-α-tubulin (CAT#ab11304, Abcam)

Figure S2c

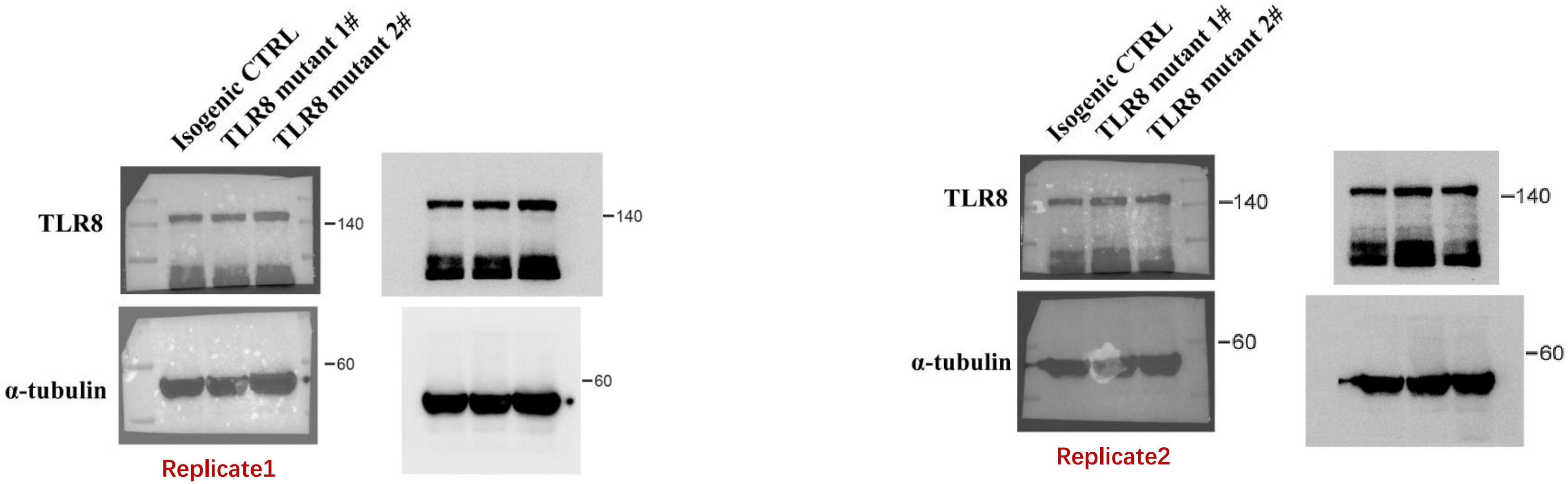

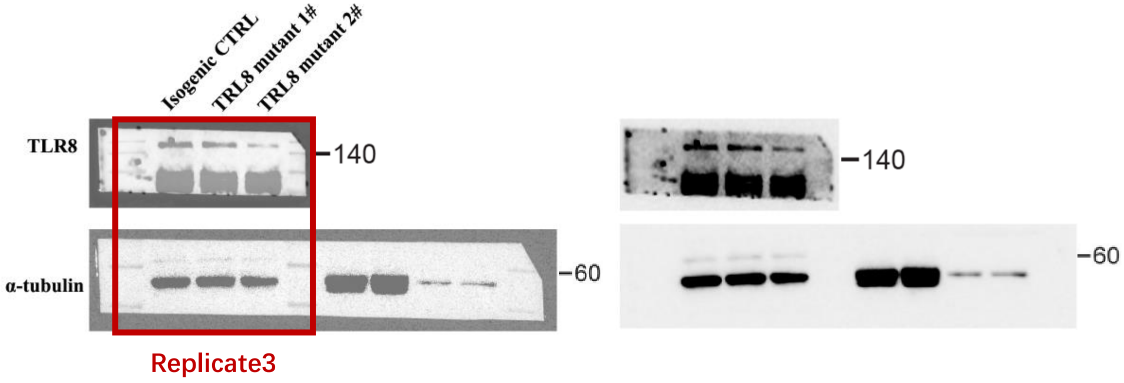

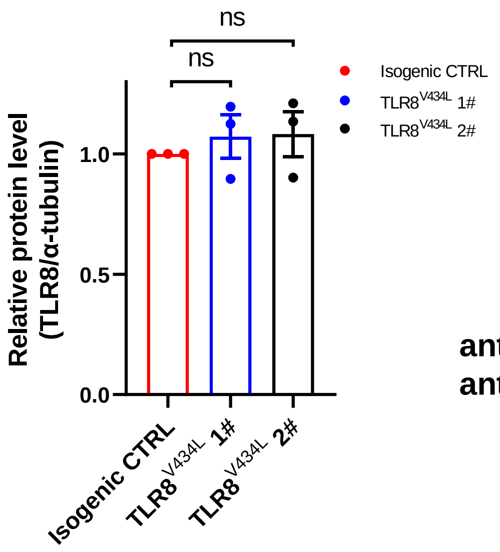

anti-TLR8 (CAT# 11886, CST)
anti-α-tubulin (CAT#ab11304, Abcam)

Isogenic CTRL			Mut 1#			Mut 2#		
1	1	1	1.124902385	1.196237133	0.896264864	1.135214023	1.210507968	0.901779852

Figure S2h

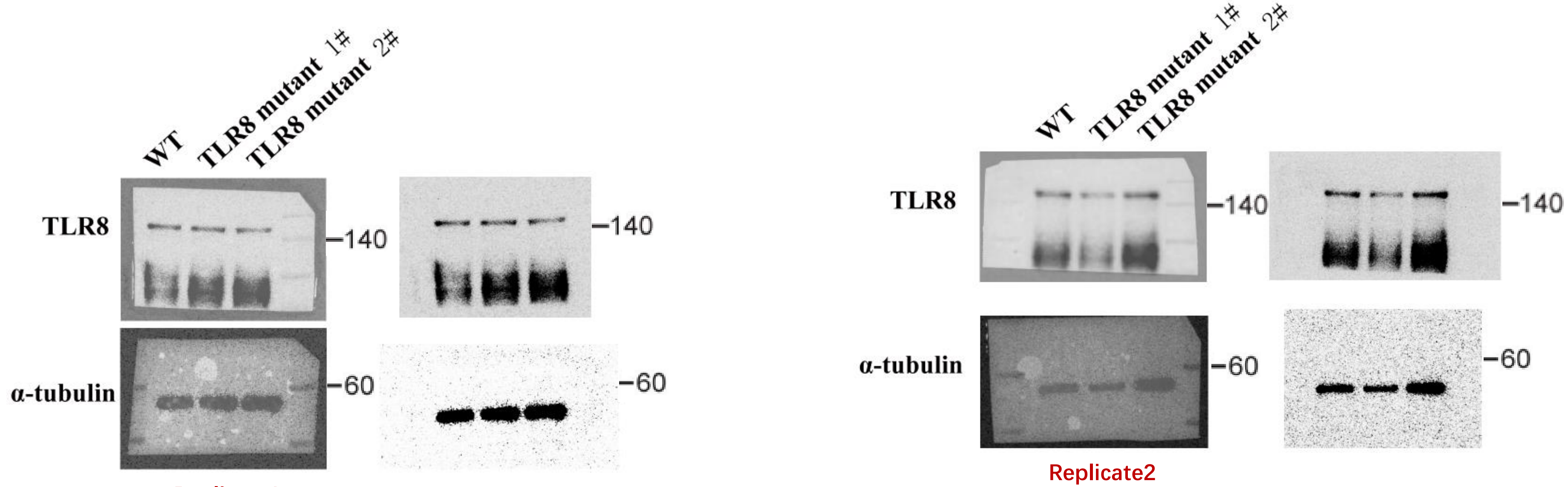

Replicate1

Replicate2

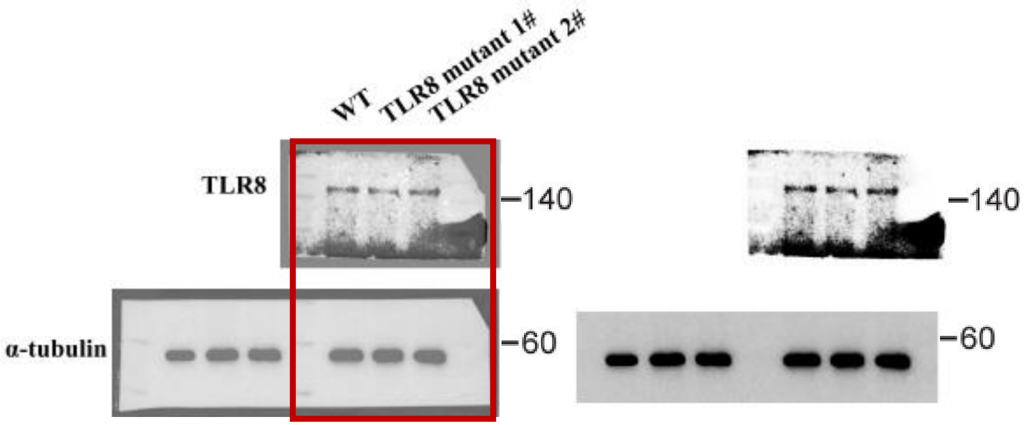

Replicate3

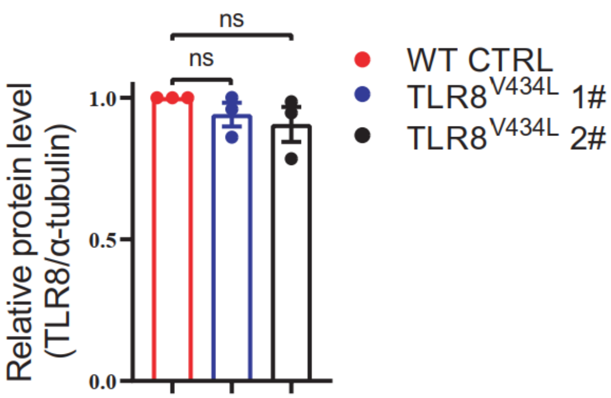

WT	mut-1	mut-2
1	0.860444645	0.985288101
1	0.959440195	0.784067004
1	1.000739003	0.946654193

anti-TLR8 (CAT# 11886, CST)
anti-α-tubulin (CAT#ab11304, Abcam)

Figure S3h

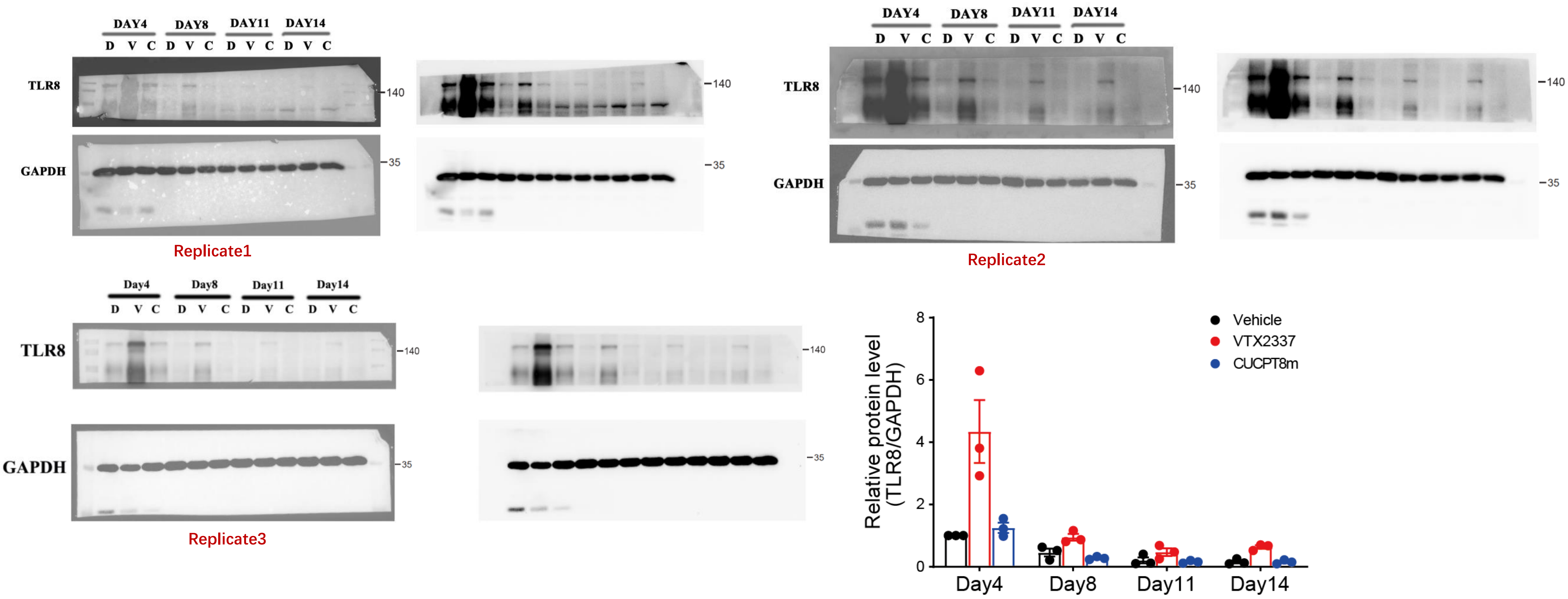

	Vehicle			VTX2337			CUCPT8m		
Day4	1	1	1	6.298583	3.811379369	2.922114776	0.984363055	1.550124901	1.222024542
Day8	0.213583085	0.524892741	0.630879224	0.813104	0.873047948	1.164161833	0.271239643	0.235500668	0.32863698
Day11	0.101477503	0.113513352	0.406070837	0.479801	0.258816943	0.684323924	0.11814987	0.154626832	0.204979697
Day14	0.094898171	0.121132829	0.254585207	0.675147	0.526053874	0.702398644	0.151140822	0.096115009	0.221060656

anti-TLR8 (CAT# 11886, CST)
anti-GAPDH (CAT# 60004-1-Ig, Proteintech)

Figure S4d

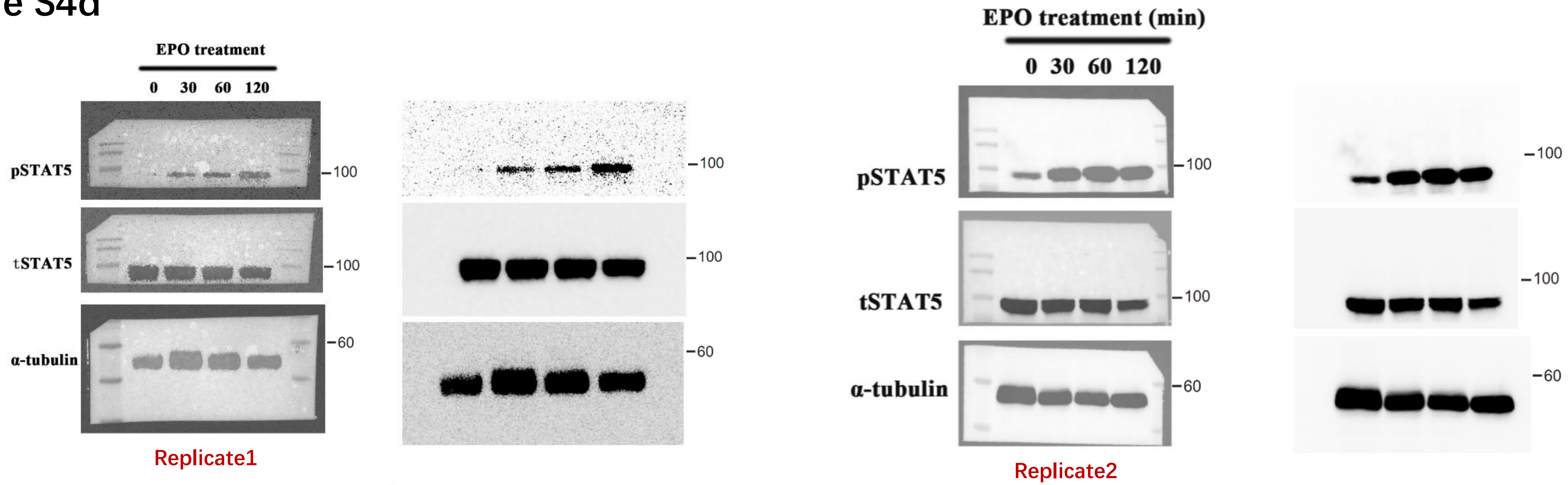

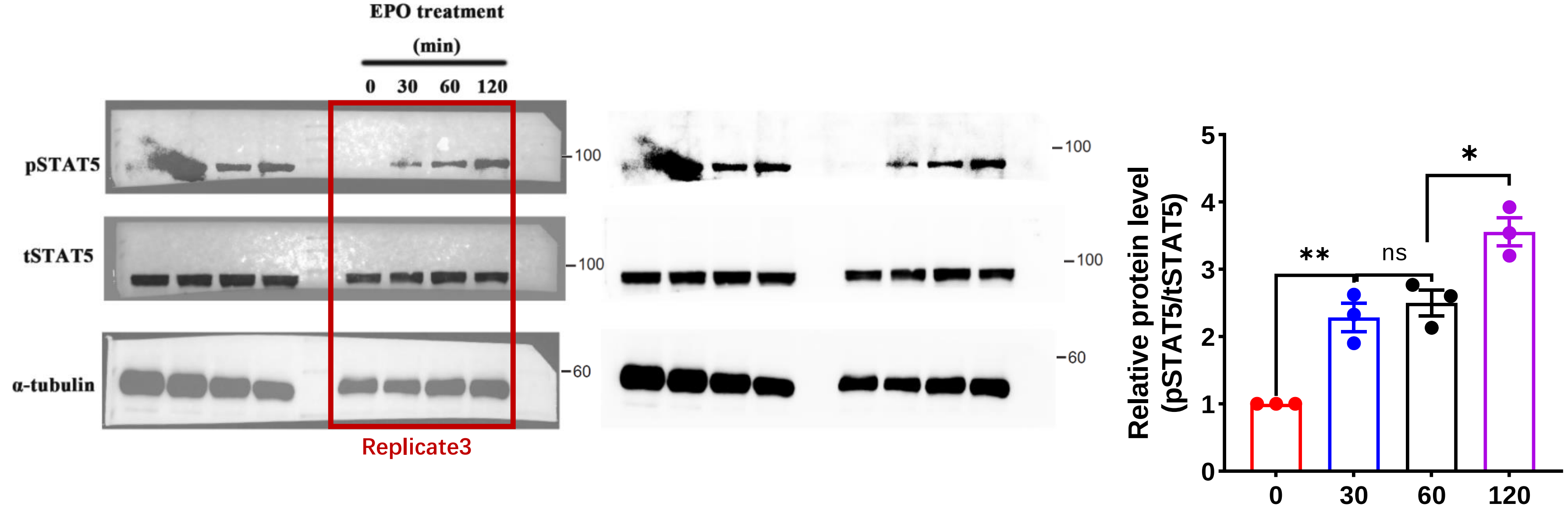

anti-p-STAT5 (CAT# 9359, CST)
anti-STAT5 (CAT# 94205, CST)
anti-α-tubulin (CAT#ab11304, Abcam)

0min			30min			60min			120min		
1	1	1	2.621734387	1.900944114	2.327629798	2.601621949	2.129742193	2.770804994	3.540038195	3.202933456	3.922914357

Figure S4e

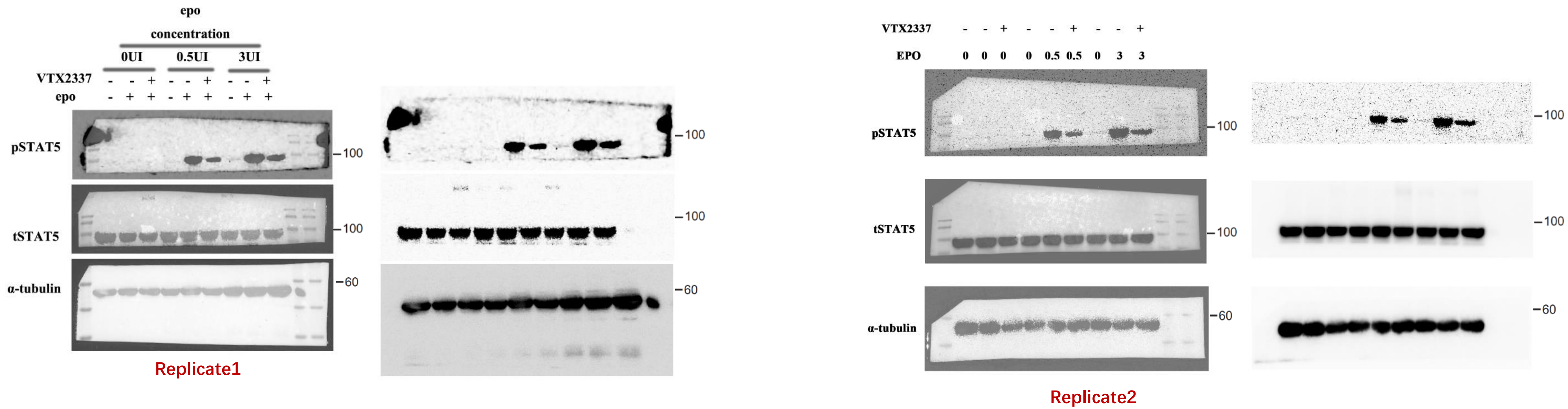

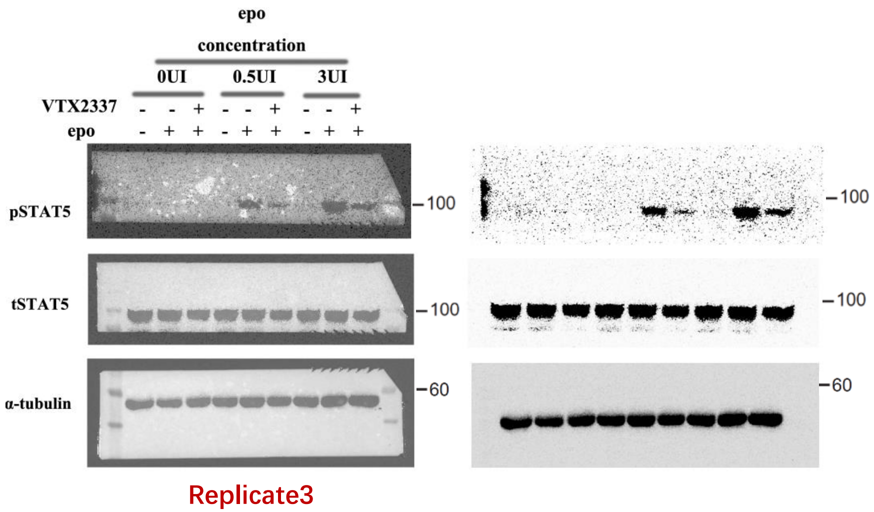

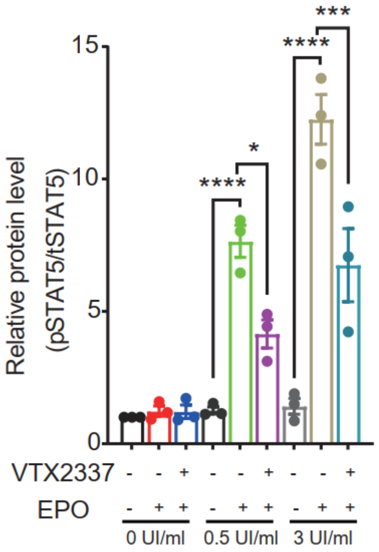

anti-p-STAT5 (CAT# 9359, CST)
anti-STAT5 (CAT# 94205, CST)
anti-α-tubulin (CAT#ab11304, Abcam)

-/-	EPO 0UI	EPO+VTX2337	-/-	EPO 0.5UI	EPO+VTX2337	-/-	EPO 3UI	EPO+VTX2337
1	1.596784701	1.024090904	1.120426472	6.455279103	3.123345278	1.508583197	10.56731017	4.22557818
1	0.928893258	0.904063144	1.140094341	8.029228316	4.898549459	0.863184781	12.41227431	7.066372329
1	1.178827645	1.715777192	1.528836111	8.449731227	4.430278178	1.890879523	13.80539942	8.959330703

Figure S4g

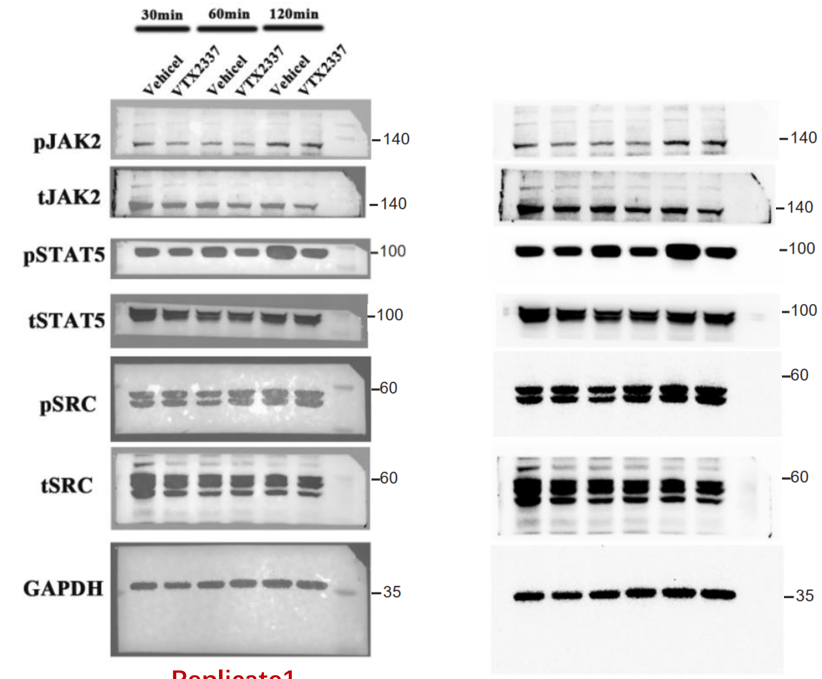

Replicate1

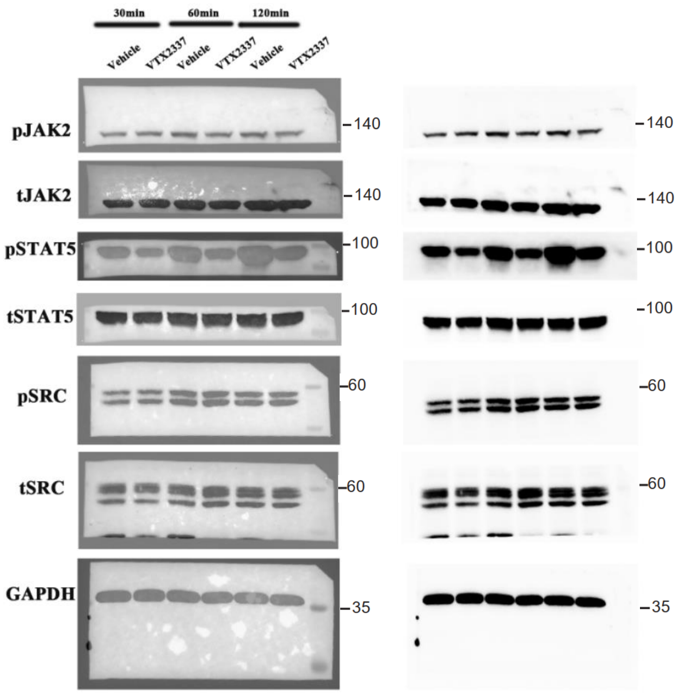

Replicate2

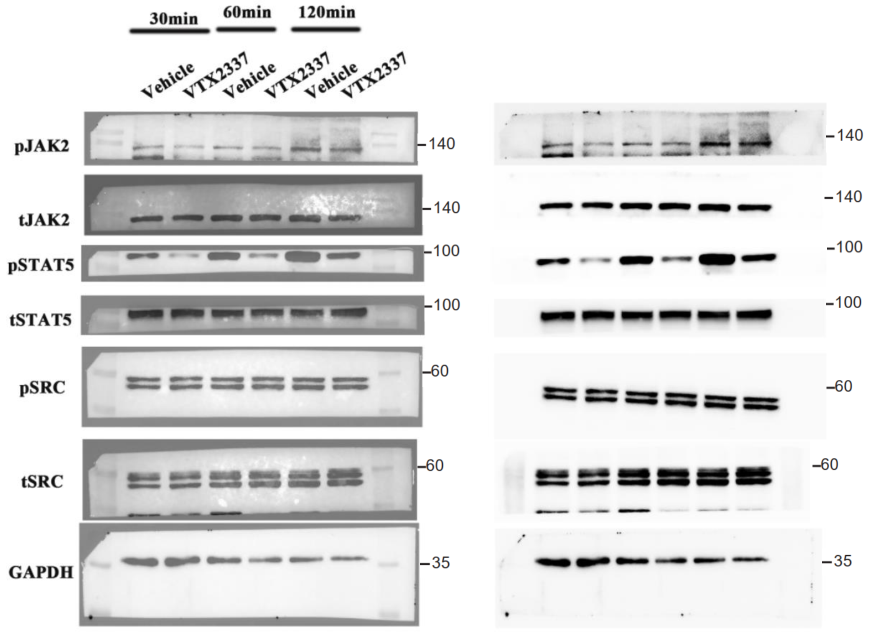

Replicate3

pstat	1	1	1	0.044598501
	0.905393566	0.720748345	0.596833423	
	1	1	1	0.022152948
	0.763251089	0.724627781	0.454553579	
	1	1	1	0.004195015
	0.678333468	0.664669675	0.469741248	
pjak	1	1	1	0.391261798
	1.017103169	0.902997051	0.982566536	
	1	1	1	0.670414557
	1.307764188	0.922731171	0.941823211	
	1	1	1	0.513008111
	1.213645559	0.834420102	1.229823658	
psrc	1	1	1	0.465109798
	1.106559318	1.09092676	0.935024053	
	1	1	1	0.379287492
	1.218875723	0.961719258	1.04402857	
	1	1	1	0.555957381
	1.027310837	0.99759724	0.902822797	

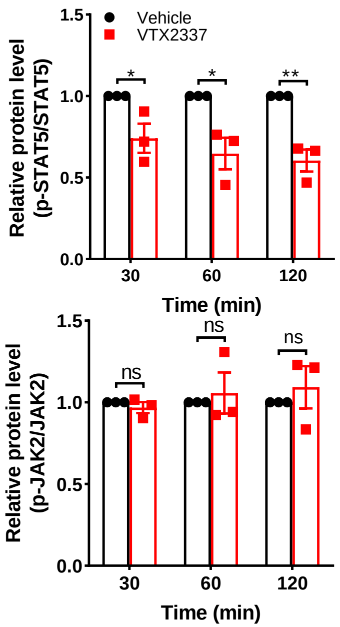

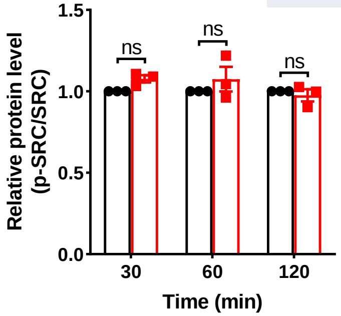

anti-p-STAT5 (CAT# 9359, CST)
anti-STAT5 (CAT# 94205, CST)
anti-JAK2 (CAT# 3230, CST)
anti-p-JAK2 (CAT# 3771, CST)
anti-SRC (CAT#110971-AP, Proteintech)
anti-p-SRC (CAT# 6943, CST)
anti-GAPDH (CAT# 60004-1-Ig, Proteintech)

Figure S4i

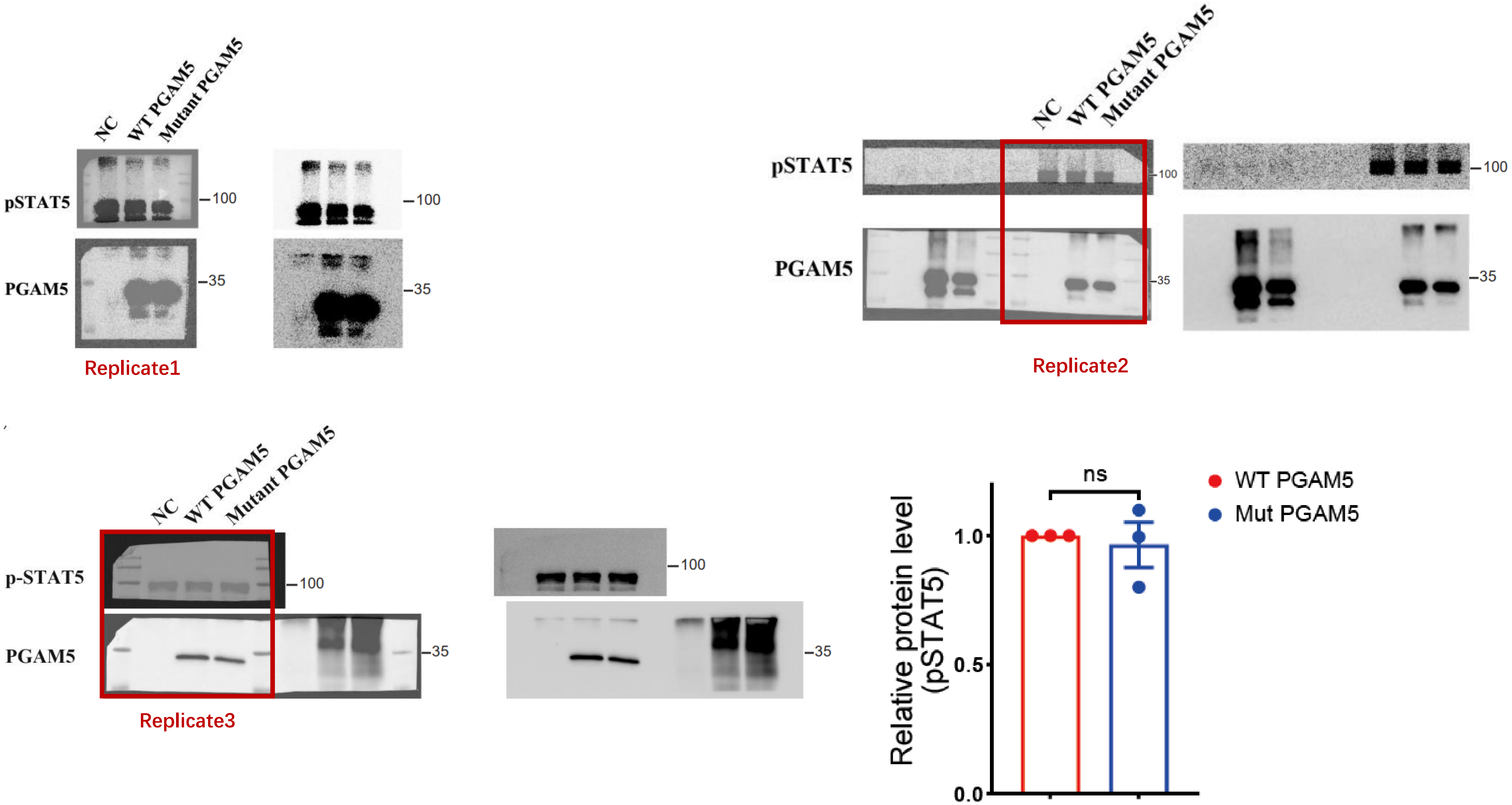

WT PGAM5			Mut PGAM5		
1	1	1	0.800407049	0.995882552	1.099026668

anti-p-STAT5 (CAT# 9359, CST)
anti-PGAM5 (CAT# 28445-1-AP, Proteintech)

Figure S4k

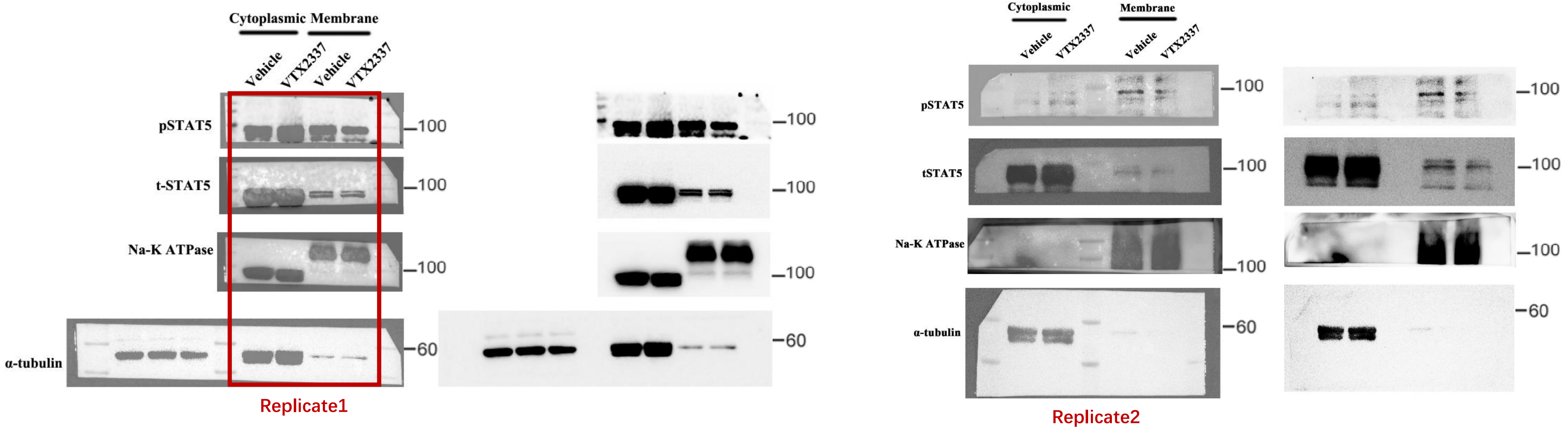

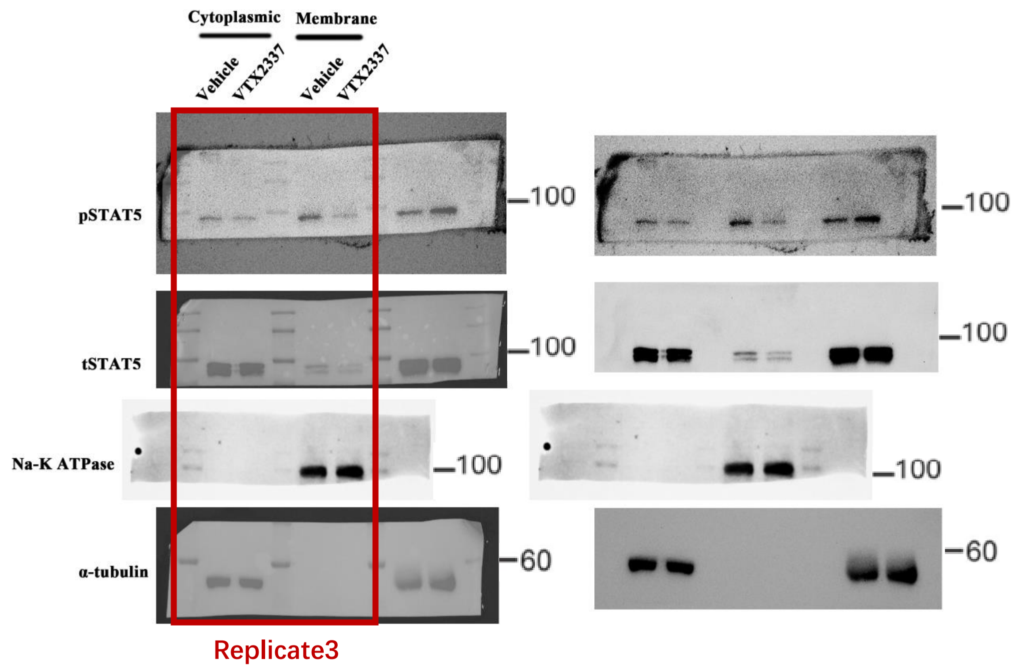

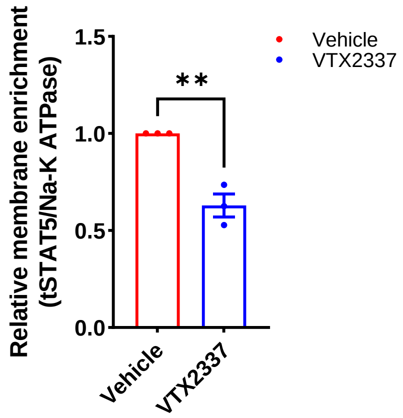

Vehicle			VTX2337		
1	1	1	0.735743193	0.527712151	0.625302898

anti-p-STAT5 (CAT# 9359, CST)
anti-STAT5 (CAT# 94205, CST)
anti-sodium potassium ATPase (CAT# ab76020, Abcam)
anti-α-tubulin (CAT#ab11304, Abcam)

Figure S4m

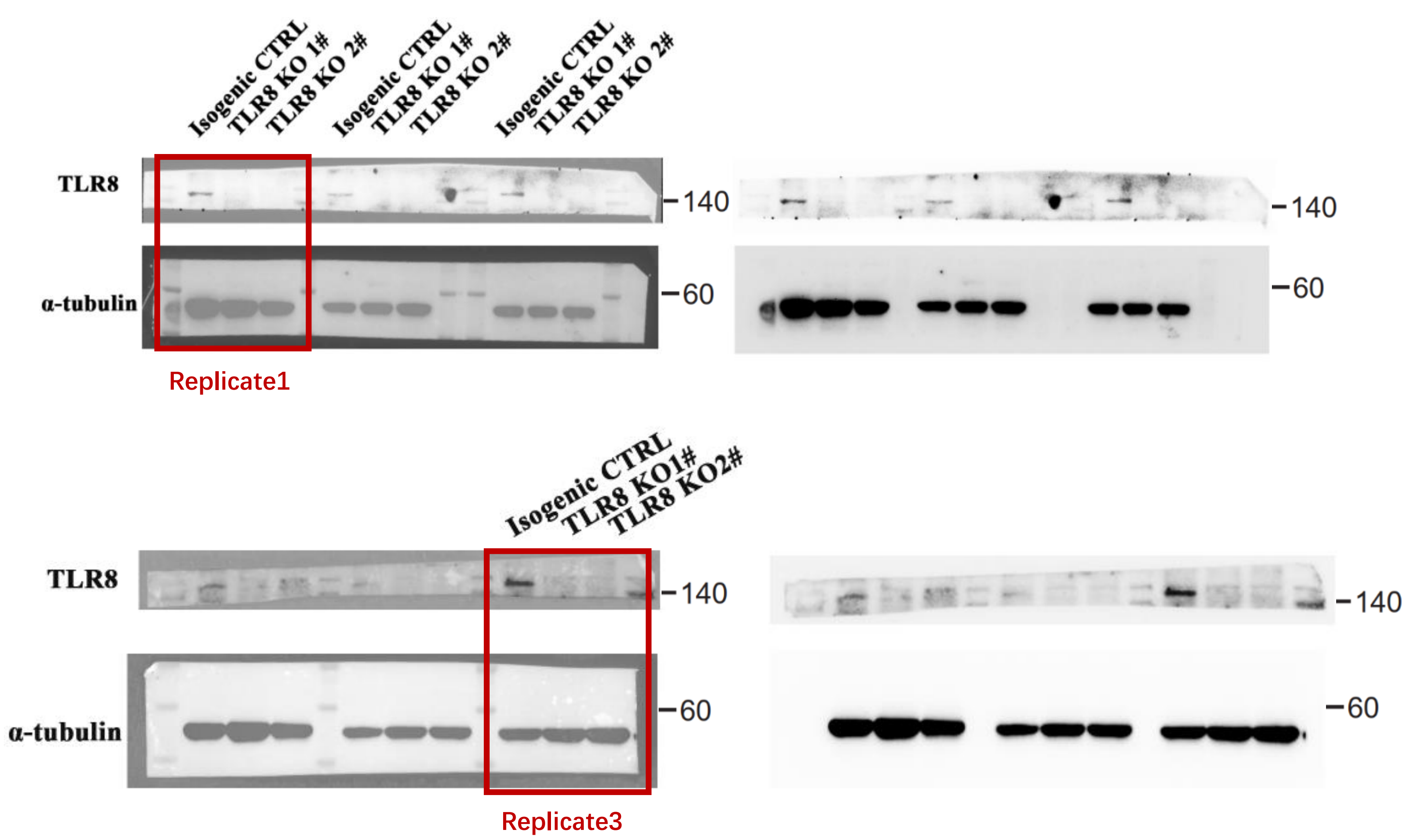

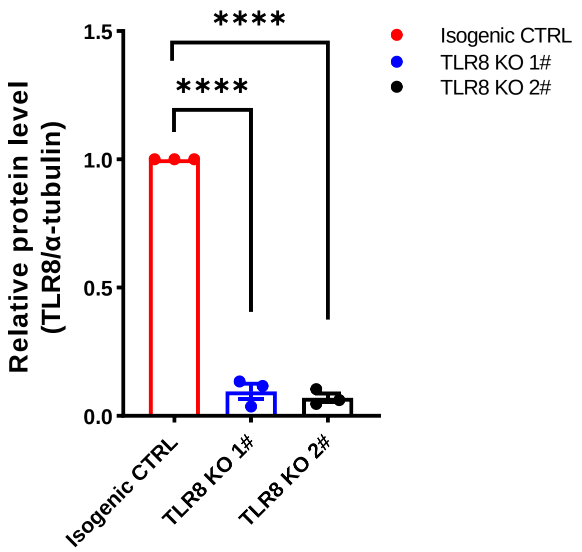

Isogenic CTRL			TLR8 KO 1#			TLR8 KO 2#		
1	1	1	0.134560981	0.037032021	0.116768134	0.104251713	0.047439917	0.061444698

anti-TLR8 (CAT# 11886, CST)
anti-α-tubulin (CAT#ab11304, Abcam)
