# Supplementary material for: Erythroid-intrinsic activation of TLR8 impairs erythropoiesis in inherited anemia
Source: Nat Commun. 2024 Jul 6;15:5678. doi: 10.1038/s41467-024-50066-w (PMC11227506; doi:10.1038/s41467-024-50066-w)
Supplement: Supplementary file 3 — Description of Additional Supplementary Files [file 41467_2024_50066_MOESM3_ESM.pdf]

Description of Additional Supplementary files

Supplementary Data 1: The list of pathogenic gene screening involved in inherited bone marrow failure syndrome. This table is provided as individual Excel file

Supplementary Data 2: Mass spectrometry analysis of proteins interact with STAT5. This table is provided as individual Excel file.
