# Supplementary material for: Erythroid-intrinsic activation of TLR8 impairs erythropoiesis in inherited anemia
Source: Nat Commun. 2024 Jul 6;15:5678. doi: 10.1038/s41467-024-50066-w (PMC11227506; doi:10.1038/s41467-024-50066-w)
Supplement: Supplementary file 6 — Reporting Summary [file 41467_2024_50066_MOESM6_ESM.pdf]

nature portfolio

Corresponding author(s): Lihong Shi

Last updated by author(s): Jun 20, 2024

Reporting Summary

Nature Portfolio wishes to improve the reproducibility of the work that we publish. This form provides structure for consistency and transparency in reporting. For further information on Nature Portfolio policies, see our Editorial Policies and the Editorial Policy Checklist.

Statistics

For all statistical analyses, confirm that the following items are present in the figure legend, table legend, main text, or Methods section.

n/a	Confirmed	
☐	☒	The exact sample size (n) for each experimental group/condition, given as a discrete number and unit of measurement
☐	☒	A statement on whether measurements were taken from distinct samples or whether the same sample was measured repeatedly
☐	☒	The statistical test(s) used AND whether they are one- or two-sided<br>*Only common tests should be described solely by name; describe more complex techniques in the Methods section.*
☐	☒	A description of all covariates tested
☒	☐	A description of any assumptions or corrections, such as tests of normality and adjustment for multiple comparisons
☐	☒	A full description of the statistical parameters including central tendency (e.g. means) or other basic estimates (e.g. regression coefficient) AND variation (e.g. standard deviation) or associated estimates of uncertainty (e.g. confidence intervals)
☐	☒	For null hypothesis testing, the test statistic (e.g. F, t, r) with confidence intervals, effect sizes, degrees of freedom and P value noted<br>*Give P values as exact values whenever suitable.*
☒	☐	For Bayesian analysis, information on the choice of priors and Markov chain Monte Carlo settings
☒	☐	For hierarchical and complex designs, identification of the appropriate level for tests and full reporting of outcomes
☒	☐	Estimates of effect sizes (e.g. Cohen's d, Pearson's r), indicating how they were calculated

Our web collection on statistics for biologists contains articles on many of the points above.

Software and code

Policy information about availability of computer code

Data collection	No software were used for data collection.
Data analysis	origin 2019; Graphpad 8.0; Image J; FlowJo X; Chimera X; Sentieon

For manuscripts utilizing custom algorithms or software that are central to the research but not yet described in published literature, software must be made available to editors and reviewers. We strongly encourage code deposition in a community repository (e.g. GitHub). See the Nature Portfolio guidelines for submitting code & software for further information.

Data

Policy information about availability of data

All manuscripts must include a data availability statement. This statement should provide the following information, where applicable:

- Accession codes, unique identifiers, or web links for publicly available datasets
- A description of any restrictions on data availability
- For clinical datasets or third party data, please ensure that the statement adheres to our policy

All experiment data are supplied in the Source Data and Supplementary Information. The raw sequence data reported in this paper have been deposited in the Genome Sequence Archive (Genomics, Proteomics & Bioinformatics 2021) in National Genomics Data Center (Nucleic Acids Res 2022), China National Center for Bioinformation / Beijing Institute of Genomics, Chinese Academy of Sciences (GSA-Human: HRA003611) that are publicly accessible at https://ngdc.cncb.ac.cn/gsa-human. The statement has been added to the Data Availability section of Methods.

Research involving human participants, their data, or biological material

Policy information about studies with human participants or human data. See also policy information about sex, gender (identity/presentation), and sexual orientation and race, ethnicity and racism.

Reporting on sex and gender	Our findings apply to an sex/gender. Also, we have obtained the information of patients with written informed consent for publication of the potentially identifiable medical data included.
Reporting on race, ethnicity, or other socially relevant groupings	All participants are Asian, Chinese, Han nationality.
Population characteristics	The proband(male,48months). DBA patient 1(RPS19mut,male, 11months), DBA patient 2(RPL5mut,14months), DBA patient 3 (RPL5mut, 22months). Healthy donor 1 (male,46yr); healthy donor 2 (female, 41yr); healthy donor 3 (male, 32yr).
Recruitment	The proband and his relatives are chosen based on the symptoms of the proband and his pedigree with family diseases.
Ethics oversight	The written informed consent for the analysis of human samples and the publication of potentially identifiable medical data was obtained from the research participants or their parents/legal guardians. The present study was approved by the Ethical Committee on Medical Research at the Institute of Hematology and Blood Diseases Hospital (Tianjin, China, KT2019090-EC-2, KT2019090-EC-3) and followed the CARE guidelines and the STROBE statement.

Note that full information on the approval of the study protocol must also be provided in the manuscript.

Field-specific reporting

Please select the one below that is the best fit for your research. If you are not sure, read the appropriate sections before making your selection.

☒ Life sciences ☐ Behavioural & social sciences ☐ Ecological, evolutionary & environmental sciences

For a reference copy of the document with all sections, see nature.com/documents/nr-reporting-summary-flat.pdf

Life sciences study design

All studies must disclose on these points even when the disclosure is negative.

Sample size	The sample sizes chosen in our study were mainly based on published studies in the same research field (Citations: DOI: 10.1038/s41421-022-00389-z and DOI: 10.3324/haematol.2022.282068) . Also, our research experience and the some samples difficult to obtain determined the sample size as well.
Data exclusions	Outlier values have been excluded and described in the manuscript or files.
Replication	Assays were repeated at least 3 times with independent biological replicates. We confirm that all replications of all assays were successful and raw data is included in the manuscript appendices.
Randomization	Samples were randomly selected for subsequent experiments.
Blinding	All quantifications and observations were performed blindingly.

Reporting for specific materials, systems and methods

We require information from authors about some types of materials, experimental systems and methods used in many studies. Here, indicate whether each material, system or method listed is relevant to your study. If you are not sure if a list item applies to your research, read the appropriate section before selecting a response.

Materials & experimental systems

n/a	Involved in the study	
☐	☒	Antibodies
☐	☒	Eukaryotic cell lines
☒	☐	Palaeontology and archaeology
☒	☐	Animals and other organisms
☒	☐	Clinical data
☒	☐	Dual use research of concern
☒	☐	Plants

Methods

n/a	Involved in the study	
☒	☐	ChIP-seq
☐	☒	Flow cytometry
☒	☐	MRI-based neuroimaging

Antibodies

Antibodies used	eFluo450-conjugated CD3 (CAT# 48003742, eBioscience, OKT3, 1:100)<br>eFluo450-conjugated CD4 (CAT# 48004942, eBioscience, RPA-T4, 1:100)<br>eFluo 450-conjugated CD14 (CAT# 48014942, eBioscience, 61D3, 1:100)<br>eFluo 450-conjugated CD19 (CAT# 48019942, eBioscience, HIB19, 1:100)<br>PE-Cy7-conjugated CD34 (CAT# 25034942, eBioscience, 4H11, 1:100)<br>BV605-conjugated CD123 (CAT# 306026, Biolegend,6H6, 1:100)<br>FITC-conjugated CD71 (CAT# 11071942, eBioscience, OKT9 (OKT-9), 1:100)<br>APC-conjugated CD235a (CAT# 551336, BD Bioscience, GA-R2, 1:100)<br>PerCP/Cy5.5-conjugated CD36 (CAT# 561536, BD Bioscience, CB38, 1:100)<br>PE-conjugated CD45 (CAT# 555483, eBioscience, HI30, 1:100)<br>APC-conjugated CD41a (CAT# 559777, BD Bioscience, HIP8, 1:100)<br>PE-conjugated TLR8 (MA516194, Thermo Fisher, 44C143, 1:50)<br>PE-isotype IgG for TLR8 (CAT# 555749, BD Bioscience, MOPC-21, 1:100)<br>PE-conjugated CD235a (CAT# 12998782, eBioscience, HIR2 (GA-R2), 1:100)<br>APC-conjugated CD71 (CAT# 17071942, eBioscience, OKT9 (OKT-9), 1:100)<br>APC-conjugated CD11b (CAT# 101212, Biolegend, M1/70, 1:100)<br>anti-EEA1,(CAT# ab70521, Abcam, 1G11, 1:200)<br>anti-TLR8, (CAT# bs-8684R, Bioss, 1:200)<br>isotype IgG for TLR8, (CAT# PP64, SigmaAldrich, 1:200)<br>anti-STAT5a, (CAT# SC-271542, Santa Cruz, C-6, 1:200)<br>488 donkey anti-mouse IgG (H+L), (CAT# A21202, Thermo Fisher, 1:200)<br>594 donkey anti-rabbit IgG (H+L), (CAT# A21207, Thermo Fisher, 1:200)<br>anti-JAK2, (CAT# 3230, CST, D2E12, 1:1000)<br>anti-p-JAK2 (CAT# 3771, CST, Tyr1007/1008, 1:1000)<br>antiSTAT5 (CAT# 94205, CST, D2O6Y, 1:1000)<br>anti-p-STAT5 (CAT# 9359, CST, C11C5, 1:1000)<br>anti-SRC (CAT# 110971-AP, Proteintech, 1:1000)<br>anti-p-SRC (CAT# 6943, CST, D49G4, 1:1000)<br>anti-α-tubulin (CAT# ab11304, Abcam, B-5-1-2, 1:5000)<br>anti-β-actin (CAT# 3700, CST, 8H10D10, 1:5000)<br>anti-GAPDH (CAT# 600041-Ig, Proteintech, 1E6D9, 1:5000)<br>anti-RPS19 (CAT# SC-100836, Santa Cruz, WW-4, 1:500)<br>anti-TLR8 (CAT# 11886, CST, D3Z6J, 1:1000)<br>anti-GATA1 (CAT# ab181544, Abcam, EPR17362, 1:2000)<br>anti-ANXA2 (CAT# 11256-1-AP, Proteintech, 1:2000)<br>anti-PGAM5 (CAT# 28445-1-AP, Proteintech, 1:2000)<br>anti-sodium potassium ATPase (CAT# ab76020, Abcam, EP1845Y, 1:2000)<br>HRP-conjugated goat anti-mouse IgG (H+L) (CAT# SA00001-1, Proteintech, 1:5000)<br>HRP-conjugated goat anti-rabbit IgG (H+L) (CAT# SA00001-2, Proteintech, 1:5000)
Validation	All primary antibodies were previously validated. For more information, please check the manufacturer's website listed below: www.thermofisher.com; www.biolegend.com; www.bdbiosciences.com; www.abcam.com; www.cellsignal.com; www.ptgcn.com; www.sigmaaldrich.com; www.scbt.com; www.bioss.com.cn. To further validate the anti-TLR8 antibodies, we used over-expressing HEK293T cells, IFN-γ treated THP-1 cells as positive controls and WT HEK293T cells as negative control. For this part of information, please check our supplementary informations.

Eukaryotic cell lines

Policy information about cell lines and Sex and Gender in Research

Cell line source(s)	THP-1 and HEK293-T cells are provided by the Management Platform of Cell Bank and originally purchased from ATCC. The male H1 human embryonic stem cells were purchased from the WiCell Research Institute (Madison, WI, USA). Cord blood primary cells were provided by Biobank at the Blood Diseases Hospital. Bone marrow samples from the patients and healthy donors were also collected at the Blood Diseases Hospital. HuDEP2 cells provided by Yukio Nakamura.
Authentication	All cell lines and primary cells were obtained and authenticated by the Management Platform of Cell Bank and Biobank at the Blood Diseases Hospital. HuDEP2 cells provided by Yukio Nakamura were authenticated by them and rechecked using our morphological assay.
Mycoplasma contamination	All cells used are Mycoplasma-negative.
Commonly misidentified lines<br>(See ICLAC register)	No commonly misidentified cell lines were used in the study.

Plants

Seed stocks	Not applicable.
Novel plant genotypes	Not applicable.
Authentication	Not applicable.

Flow Cytometry

Plots

Confirm that:

☒ The axis labels state the marker and fluorochrome used (e.g. CD4-FITC).

☒ The axis scales are clearly visible. Include numbers along axes only for bottom left plot of group (a 'group' is an analysis of identical markers).

☒ All plots are contour plots with outliers or pseudocolor plots.

☒ A numerical value for number of cells or percentage (with statistics) is provided.

Methodology

Sample preparation	Cells were washed with PBS and centrifuged at 300 g for 5 minutes. The supernatant was then discarded and the cells were re-suspended in 100 µl FACS buffer (PBS with 2% FBS + 2 mM EDTA). Cells were incubated with antibodies (diluted at 1:100) at 4°C in the dark for 30 minutes and washed by and resuspended in FACS buffer for flow cytometric analysis (1 ~ 5x105) or sorting (1 ~ 5x107). BFU-E and CFU-E were immunophenotyped as previously reported. Erythroid precursors were defined as CD71+CD235a+ cells.
Instrument	FACSAria III (BD Biosciences), FACS LSRII (BD Biosciences)
Software	FlowJo V10
Cell population abundance	Cell populations were sorted to >80% purity. Purity was determined by analyzing sorted cells with colony-forming assay.
Gating strategy	Gates were set manually by using isotype IgG and appropriate control samples. Example gating strategies are provided in Figure 3d.

☐ Tick this box to confirm that a figure exemplifying the gating strategy is provided in the Supplementary Information.
